# Integrated taxonomy reveals new threatened freshwater mussels (Bivalvia: Hyriidae: *Westralunio*) from southwestern Australia

**DOI:** 10.1038/s41598-022-24767-5

**Published:** 2022-11-27

**Authors:** Michael W. Klunzinger, Corey Whisson, Alexandra Zieritz, Justin A. Benson, Barbara A. Stewart, Lisa Kirkendale

**Affiliations:** 1 Australian Rivers Institute, Griffith University, Nathan, QLD 6111 Australia; 2 Mollusc Section, Department of Aquatic Zoology, Western Australian Museum, Welshpool, WA 6163 Australia; 3 School of Geography, University of Nottingham, University Park, Nottingham, NG7 2RD UK; 4 Centre for Natural Resource Management, UWA School of Agriculture and the Environment, The University of Western Australia, Albany, WA 6330 Australia

**Keywords:** Molecular ecology, Taxonomy, DNA sequencing, RNA sequencing, Conservation biology

## Abstract

The freshwater mussel *Westralunio carteri* (Iredale, 1934) has long been considered the sole *Westralunio* species in Australia, limited to the Southwest and listed as vulnerable on the IUCN Red List and under Australian legislation. Here, we used species delimitation models based on COI mtDNA sequences to confirm existence of three evolutionarily significant units (ESUs) within this taxon and conducted morphometric analyses to investigate whether shell shape differed significantly among these ESUs. “*W. carteri*” I was found to be significantly larger and more elongated than “*W. carteri*” II and “*W. carteri*” II + III combined, but not different from “*W. carteri*” III alone. We recognise and redescribe “*W. carteri*” I as *Westralunio carteri* (Iredale, 1934) from western coastal drainages and describe “*W. carteri*” II and “*W. carteri*” III as *Westralunio inbisi* sp. nov. from southern and lower southwestern drainages. Two subspecies are further delineated: “*W. carteri*” II is formally described as *Westralunio inbisi inbisi* subsp. nov. from southern coastal drainages, and “*W. carteri*” III as *Westralunio inbisi meridiemus* subsp. nov. from the southwestern corner. Because this study profoundly compresses the range of *Westralunio carteri* northward and introduces additional southern and southwestern taxa with restricted distributions, new threatened species nominations are necessary.

## Introduction

There has been a growing interest in using multiple lines of evidence for delineating species boundaries, particularly for taxa such as freshwater mussels, which are highly threatened while containing undescribed cryptic diversity^1–8^. Before the advent of modern molecular systematics and taxonomy, new freshwater mussel species were described based primarily on shell morphology and morphometry^9–11^. However, recognition of freshwater mussel species based solely on morphology can be fraught with difficulties, with freshwater mussel taxonomy hindered, in part, by the tendency for shell forms to vary in response to environmental conditions^12–15^.

Freshwater mussels (Bivalvia: Palaeoheterodonta: Unionida) are comprised of six families with a total of 192 genera and 958 species worldwide^16^. Six unionidan families are recognised as distinct based on larval morphology, the number and arrangement of ctenidia demibranchs containing marsupia, water tube and brood chamber morphology in the demibranchs, the presence or absence of a supra-anal aperture and mantle fusion relative to the inhalant and exhalant siphons^17,18^. Most freshwater mussels have a two-stage life cycle, whereby larvae are briefly parasitic on fishes and, in some cases, amphibians for a brief period of weeks to months and live the remainder of their lives as benthic filter-feeders. Larval forms include glochidia in the Hyriidae, Margaritiferidae and Unionidae and lasidia or haustoria in Etheriidae, Iridinidae and Mycetopodidae^19^.

The Hyriidae have a Gondwanan origin and include at least 96 species from 16 genera with a trans-Pacific distribution in Australasia and South America^14,20–23^. Included amongst these genera is the freshwater mussel genus *Westralunio*. This genus is restricted to the Australasian region in the Southern Hemisphere and is represented by three species: *Westralunio carteri* (Iredale, 1934^9^) from southwestern Australia, *Westralunio flyensis* (Tapparone Canefri, 1883^24^) from Papua New Guinea and *Westralunio albertisi* Clench, 1957^25^ from Papua New Guinea and eastern Indonesian West Papua^14^. Iredale^9^ erected the genus *Westralunio* based on hinge dentition, and established two subspecies, *Westralunio amibiguus ambiguus* (Philippi, 1847^26^) and *Westralunio ambiguus carteri* Iredale, 1934^9^ based on geography and shell form. McMichael & Hiscock^10^ later merged the two subspecies, stating that they could not be separated on anatomical or geographical grounds and elevated the name *carteri* to specific rank, ceding the name *ambiguus* to the genus *Velesunio* from eastern Australia. As such, *W. carteri* was recognised as the sole southwestern Australian freshwater mussel. McMichael & Hiscock^10^ also included *W. flyensis* and *W. albertisi* in the genus, stating that “*Westralunio* is, in most respects, a typical velesunionine mussel, but it is easily distinguished from the related genera, *Velesunio* and *Alathyria*, by its strong, grooved cardinal teeth.”

Two recent studies have investigated phylogeographic structuring and the existence of Evolutionary Significant Units (ESUs) and Molecular Operational Taxonomic Units’ (MOTUs) in *W. carteri*^27,28^*.* Klunzinger et al.^27^ applied four species delimitation models based on 46 COI mtDNA sequences spanning 13 populations of *W. carteri,* which revealed unanimous support for at least two MOTUs: “*W. carteri*” I from west coast drainages and “*W. carteri*” II + III from drainages of the south coast lower southwest of southwestern Australia. The degree of differentiation (2.8–3.4%) between these two putative MOTUs and their apparent allopatry, led these authors to suggest that they be recognised as distinct species. Furthermore, one of the four models revealed a potential third MOTU (“*W. carteri*” III), comprising individuals from the southwest corner of the region. The authors recommended further research was required to better characterise the observed differentiation among lineages and their distribution. Subsequent work by Benson et al.^28^ incorporated COI and 16S sequences from an additional 119 individuals from 19 populations previously unsampled and showed that the distribution of the two primary lineages rarely overlapped, and that the third lineage appeared to be restricted to just two river systems.

These two independent lines of evidence (genetics and geographical distribution) lend support to the notion that these three *Westralunio* lineages warrant species-level consideration and formal taxonomic description which, to date, has not been undertaken. Furthermore, *W. carteri* also exhibits marked intraspecific variation in shell morphology, with specimens collected from locations on the south coast^29^ appearing to be less elongate and having more squarely truncated posteriors than those from other parts of the species’ range (M.W. Klunzinger, pers. obs.).

As species are part of the fundamental unit of conservation assessment^30^, an integrated taxonomic evaluation is required for *W. carteri*, incorporating analyses of morphological variation and the application of species delimitation models to the full data set (i.e., combining Klunzinger et al*.*^27^ and Benson et al*.*^28^). However, this has yet to be undertaken.

The purpose of this study was to revisit the taxonomy of *W. carteri* based on an integrative taxonomic approach with a view to test the hypotheses that *W. carteri* either represents a geographically variable, single species or consists of multiple taxa worthy of taxonomic recognition. More specifically, we (i) used three species delimitation models and a comprehensive data set of COI mtDNA sequences to confirm the existence of previously identified ESUs, (ii) tested whether these taxa are morphologically distinct, and (iii) formally described the ESUs recognised for *W. carteri* as separate taxa.

## Results

### Genetic variation

**Figure 1:**
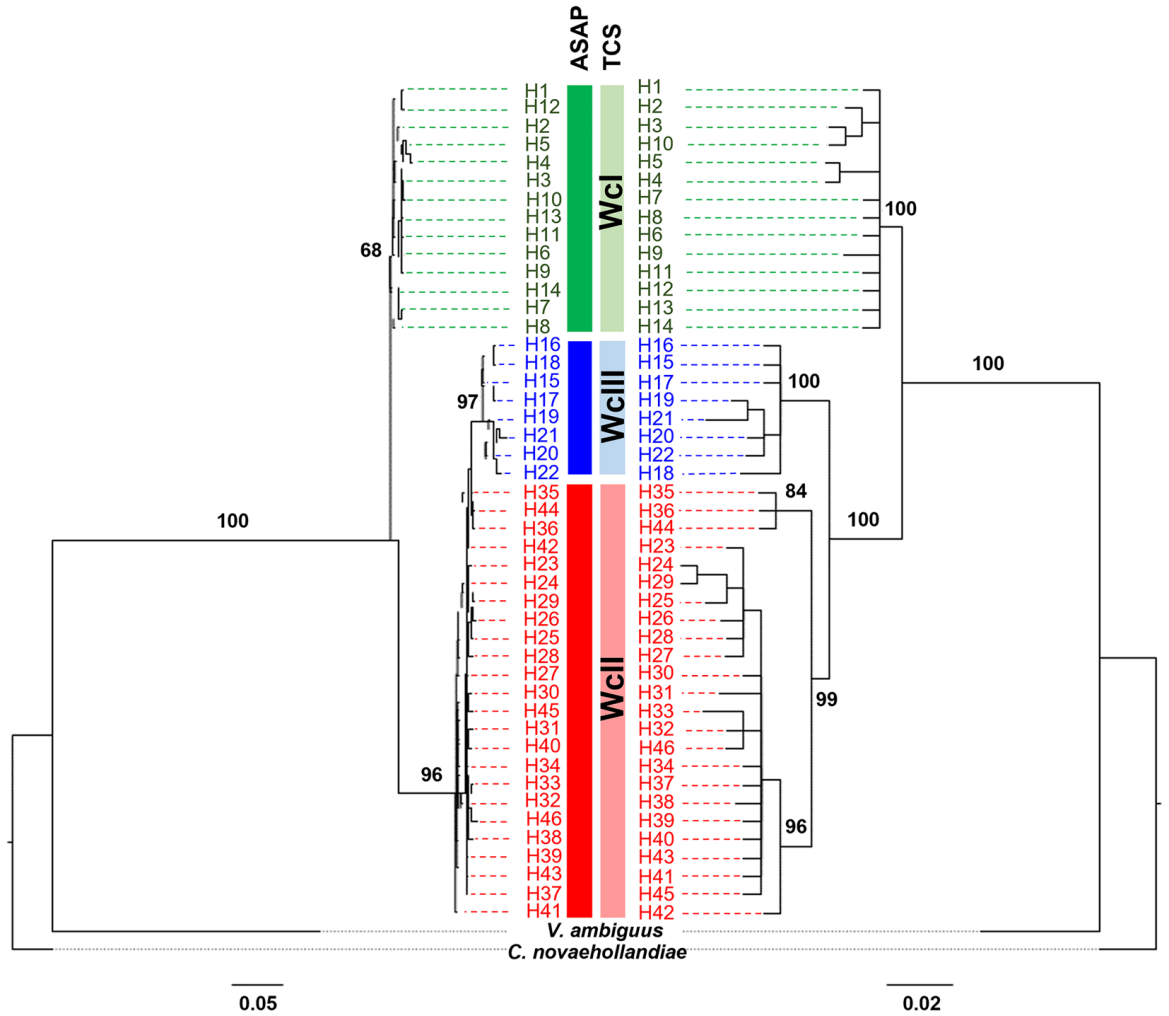
Phylogenetic trees obtained by maximum likelihood (left) and Bayesian inference (right) analysis of “*Westralunio carteri*” mtDNA COI sequences, including support values for the major genetic clades [ultrafast bootstrap values (left) and Bayesian posterior probabilities (right)]. Colour coded bars show support for the three major clades by the species delimitation methods (ASAP = dark shade; TCS = lighter shade). Green = WcI = “*W. carteri*” I; blue = WcIII = “*W. carteri*” III; red = WcII = “*W. carteri*” II. Results of bPTP analysis not shown (see supplementary data). Haplotype names correspond to Benson et al.^28^. Outgroup taxa are *Velesunio ambiguus* (Philippi, 1847) (Hyriidae: Velesunioninae) and *Cucumerunio novaehollandiae* (Gray, 1834) (Hyriidae: Hyriinae: Hyridellini).

The best fitting substitution models for COI codons 1–3 were identified as TN + F + G4, F81 + F + I, and TN + F, respectively. The maximum likelihood (ML) and Bayesian inference (BI) trees showed similar topologies of the main nodes, although the BI tree displayed greater resolution of the ingroup branches (Fig. 1). Furthermore, the BI tree revealed three monophyletic clades, while two of those clades were merged in the ML tree. Two of the three molecular species delimitation methods (ASAP and TCS) recovered three groups in the BI tree as distinct taxa (Fig. 1), corresponding to the three previously described ESUs^27,28^. The third method (bPTP) recovered between 8 and 43 groups (mean = 28.03) suggesting that there is evidence of additional genetic differentiation within the three groups identified by ASAP and TCS. The outputs of the three methods are provided in the Supplementary information. The molecular diagnosis uncovered several fixed nucleotide differences COI characters for each taxon (Table 1: “*W. carteri*” I = 10; “*W. carteri*” II = 3; “*W. carteri*” III = 5). There were also 13 fixed nucleotide differences in *W. carteri* for the 16S gene. The remaining two taxa had no fixed nucleotide differences for the 16S gene.

**Table 1:** Molecular diagnoses of “*Westralunio carteri*” Evolutionarily Significant Units (ESUs) from southwestern Australia (after Bolotov et al.^122﻿^ with reanalysis of data from Klunzinger et al*.*^27^ and Benson et al*.*^28^).

ESU/taxon	Number of samples (COI/16S)	Mean COI P-distance from nearest neighbour of new species, % and (SE)	Nearest neighbour of new species (COI)	Mean 16S P-distance from nearest neighbour of new species, % and (SE)	Nearest neighbour of new species (16S)	Diagnostic characters based on the sequence alignment of congeners	
						COI	16S
“*W. carteri*” I	60/40	5.02 (0.90)	“*W. carteri*” II	2.95 (0.01)	“*W. carteri*” II	57 G, 117 T, 210 G, 249 T, 255 C, 345 G, 423 T, 447 T, 465 A, 499 T	137 T, 155 C, 228 C, 229 T, 260 G, 290 A, 305 G, 307 T, 310 A, 311 C, 321 T, 330 A, 460 A
“*W. carteri*” II	92/81	2.74 (0.62)	“*W. carteri*” III	0.57 (0.29)	“*W. carteri*” III	75 A, 87 T, 318 T	None
“*W. carteri*” III	12/9	2.74 (0.62)	“*W. carteri*” II	0.57 (0.29)	“*W. carteri*” II	69 C, 123 C, 126 T, 483 A, 526 A	None

New taxa include “*W. carteri*” II and III. Supplementary files 1 and 2 contain the alignments used to determine the single pure characters and p-distances. The position of each diagnostic character refers to its location within those alignments. SE, standard error.

### Variation in shell morphology

Based on results from analyses of variances (ANOVAs), shells of “*W. carteri*” I were significantly larger (for size metrics total length (TL), maximum height (MH), beak height (BH) and beak length (BL)) and more elongated (i.e., had a lower maximum height index (MHI)) than shells of “*W. carteri*” II and “*W. carteri*” II + III combined (Table 2). However, there was no difference in size or shape metrics between “*W. carteri*” I and “*W. carteri*” III (Table 2). The lack of significant differences in beak height index (BHI) and beak length index (BLI) among any of the taxa (Table 2) indicates that wing and anterior shell development was not discernibly different between any of the ESUs.

**Table 2:** Shell size metrics [mm], shape indices [%] and scores for the first two principal components (PC) obtained by Principal Component Analysis of shape indices and 18 Fourier coefficients generated by Fourier Shape Analysis for each “*Westralunio carteri*” species and subspecies-level Evolutionarily Significant Units (ESUs): *n*, number of specimens measured; minimum (min) to maximum (max) and mean (± standard error (SE)).

Variable	“*W. carteri*” I min–max	Mean ± SE	*n*	“*W. carteri*” II min–max	Mean ± SE	*n*	“*W. carteri*” III min–max	Mean ± SE	*n*	df	F	P
TL	12.00–92.00	59.79^B^ ± 0.68	294	28.38–79.00	51.68^A^ ± 0.90	140	45.00–84.00	58.46^AB^ ± 2.89	12	443	24.5	** < 0.0001**
MH	8.50–59.00	37.25^B^ ± 0.39	294	18.56–52.00	33.91^A^ ± 0.57	140	32.00–50.00	37.92^AB^ ± 1.34	12	443	12.33	** < 0.0001**
BH	7.00–54.00	33.88^B^ ± 0.39	294	14.72–49.00	30.63^A^ ± 0.55	140	28.00–46.00	34.04^AB^ ± 1.37	12	443	11.87	** < 0.0001**
BL	4.00–30.00	19.04^B^ ± 0.24	294	9.00–28.00	16.14^A^ ± 0.29	140	13.00–26.00	17.75^AB^ ± 1.01	12	443	26.78	** < 0.0001**
MHI	0.46–0.89	0.63^A^ ± 0.00	294	0.55–0.74	0.66^B^ ± 0.00	140	0.60–0.71	0.65^AB^ ± 0.01	12	443	28.23	** < 0.0001**
BHI	0.76–1.04	0.91^A^ ± 0.00	294	0.79–0.99	0.90^A^ ± 0.00	140	0.84–0.93	0.90^A^ ± 0.01	12	443	1.039	0.355
BLI	0.22–0.49	0.32^A^ ± 0.00	294	0.23–0.51	0.32^A^ ± 0.00	140	0.28–0.35	0.30^A^ ± 0.01	12	443	1.775	0.171
PC1 (indices)	− 3.94–6.36	− 0.04^A^ ± 0.07	294	− 2.37–5.10	0.11^A^ ± 0.10	140	− 1.43–1.11	− 0.24^A^ ± 0.20	12	443	1.058	0.348
PC2 (indices)	− 4.04–3.87	− 0.21^B^ ± 0.06	294	− 1.87–2.85	0.40^A^ ± 0.07	140	− 1.00–1.80	0.43^AB^ ± 0.25	12	443	17.61	** < 0.0001**
PC1 (Fourier)	− 0.079–0.047	0.003^B^ ± 0.001	273	− 0.057–0.048	− 0.007^A^ ± 0.002	126	− 0.027–0.032	− 0.001^AB^ ± 0.006	12	408	12.2	** < 0.0001**
PC2 (Fourier)	− 0.048–0.062	0.003^B^ ± 0.001	273	− 0.047–0.035	− 0.006^A^ ± 0.001	126	− 0.032–0.021	− 0.004^AB^ ± 0.004	12	408	12.06	** < 0.0001**

Variable	“*W. carteri*” II + III min–max	Mean ± SE	*n*	df	F	P						
TL	28.38–84.00	52.22 ± 0.87	152	444	44.73	** < 0.0001**						
MH	18.56–52.00	34.23 ± 0.54	152	444	20.52	** < 0.0001**						
BH	14.72–49.00	30.90 ± 0.52	152	444	20.64	** < 0.0001**						
BL	9.00–28.00	16.27 ± 0.28	152	444	51.54	** < 0.0001**						
MHI	0.55–0.74	0.66 ± 0.00	152	444	56.48	** < 0.0001**						
BHI	0.79–0.99	0.90 ± 0.00	152	444	1.833	0.176						
BLI	0.23–0.51	0.31 ± 0.00	152	444	2.442	0.119						
PC1 (indices)	− 2.37–5.10	0.08 ± 0.10	152	444	1.131	0.288						
PC2 (indices)	− 1.87–2.85	0.40 ± 0.07	152	444	35.29	** < 0.0001**						
PC1 (Fourier)	− 0.057–0.048	− 0.006 ± 0.001	138	409	23.32	** < 0.0001**						
PC2 (Fourier)	− 0.047–0.035	− 0.006 ± 0.001	138	409	23.91	** < 0.0001**						

P-value (P), degrees of freedom (df) and F-ratio (F) were obtained from ANOVAs comparing “*W. carteri*” I, II and III (upper section of the Table) and “*W. carteri*” I and II + III (lower section of the Table), respectively. P*-*values < 0.05 in bold; different superscript letters indicate significant differences between groups in Tukey’s pairwise posthoc-test. Shell size metrics: *TL* total length, distance from anterior to posterior apices of valve; *MH* maximum height, distance from ventral edge to apex of posterior ridge; *BH* beak height, distance from ventral edge to beak apex; *BL* beak length, distance from anterior apex of valve to the 90-degree vertex aligning with the beak apex. Shape indices determined as: MHI = MH/TL, BHI = BH/MH, and BLI = BL/TL.

**Figure 2:**
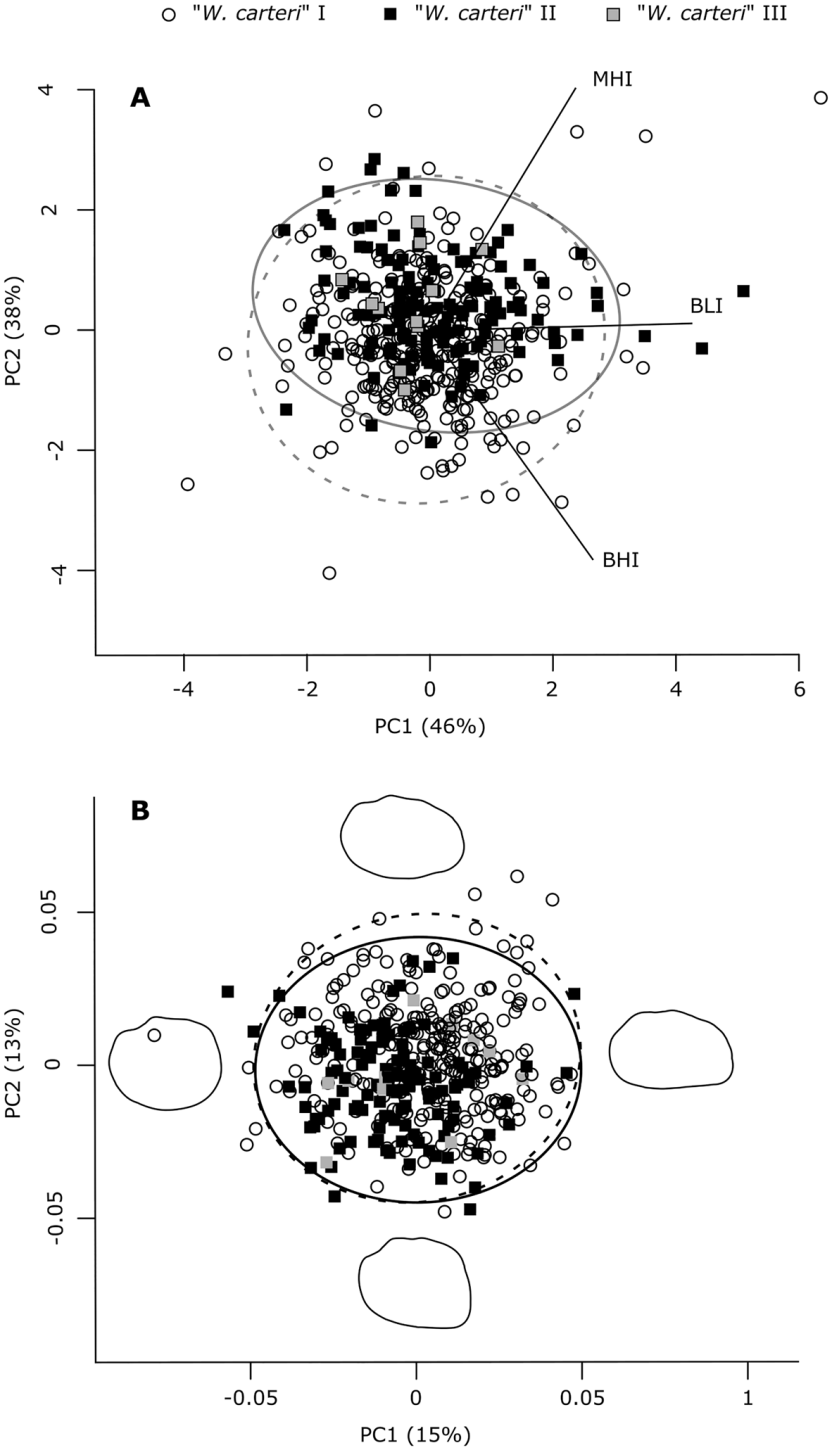
Scatterplots of the first two PC axes obtained by PCA on (**A**) calculated shape indices based on shell measurements, and (**B**) 18 Fourier coefficients for “*Westralunio carteri*” I, “*W. carteri*” II and “*W. carteri*” III. 95% Confidence Intervals are displayed at the species level, i.e., for “*W. carteri*” I (full line) and “*W. carteri*” II + III (dashed line). Extreme shell outlines in (**B**) are depicted to visualise trends in sagittal shell shape, along PC axes.

This pattern was partly confirmed in the principal component analysis (PCA) of these three shell shape indices, where PC1, largely explained by variation in BLI (Fig. 2A), did not differ between the two species (i.e., “*W. carteri*” I vs. “*W. carteri*” II + III) or among the three taxa (Table 2). The PC2, largely explained by variation in MHI and BHI (Fig. 2A), differed significantly between “*W. carteri*” I and “*W. carteri*” II (Table 2). Accordingly, 70% (70% jack-knifed) of specimens were assigned to the correct species in the corresponding discriminant analysis (DA), whilst this was true for only 55% (54%) at the MOTU-level.

The difference in shell elongation between “*W. carteri*” I and “*W. carteri*” II was confirmed by Fourier shape analysis. As visualised by synthetic outlines in Fig. 2B, shell elongation is expressed along the PC1 (explaining 15% of total variation in Fourier coefficients). The PC1 as well as PC2 scores differed significantly between the two species (i.e., “*W. carteri*” I vs. “*W. carteri*” II + III) as well as between “*W. carteri*” I and “*W. carteri*” II, respectively (Table 2). Combined with synthetic outlines, this indicated a tendency towards a more elongated, somewhat wedge-shaped shell in “*W. carteri*” I, whilst “*W. carteri*” II shells tended to be relatively high with a stout anterior margin (Fig. 2B). An analysis of similarities (ANOSIM) analysis on all Fourier coefficients revealed no significant difference between the two species (i.e., “*W. carteri*” I vs. “*W. carteri*” II + III; ANOSIM: R = − 0.018, p = 0.097), but did indicate a significant difference between the three ESUs (ANOSIM: R = 0.0625, p = 0.0051). Specifically, “*W. carteri*” I differed significantly from “*W. carteri*” II (Bonferroni-corrected p = 0.0009). Only 66% and 65% (62% and 62% jack-knifed) of specimens were assigned to the correct species and taxon in DAs on that dataset, respectively.

### Taxonomic accounts

**Class:** Bivalvia Linnaeus, 1758^31^.

**Subclass:** Autobranchia Grobben, 1894^32^.

**Infraclass:** Heteroconchia Gray, 1854^33^.

**Cohort:** Palaeoheterodonta Newell, 1965^34^.

**Order:** Unionida Gray, 1854^33^ in Bouchet & Rocroi, 2010^35^.

**Superfamily:** Unionoidea Rafinesque, 1820^36^.

**Family:** Hyriidae Parodiz & Bonetto 1963^37^.

**Genus: ***Westralunio* Iredale, 1934^9^.

**Type species:** *Westralunio ambiguus carteri* Iredale, 1934^9^ (by original designation).

### Redescription: *Westralunio carteri* (Iredale, 1934)

#### Synonymy

*Unio australis* Lamarck^38^: Menke^39^, p. 38, specimen 219. (Non *Unio australis* Lamarck, 1819^38^).

*Unio moretonicus* Reeve^40^: Smith^41^, p. 3, pl. iv, Fig. 2. (misidentified reference to *Unio moretonicus* Reeve, 1865^40^).

*Hyridella australis* (Lam.^38^): Cotton & Gabriel^42^ (in part), p. 156. (misidentified reference to *Unio australis* Lamarck, 1819^38^).

*Hyridella ambigua* (Philippi^26^): Cotton & Gabriel^42^ (in part), p. 157. (misidentified reference to *Unio ambiguus* Philippi, 1847^26^).

*Westralunio ambiguus carteri*: Iredale, 1934^9^, p. 62.

*Westralunio ambiguus* (Philippi^26^): Iredale^9^, p. 62, pl. iii, Fig. 8, pl. iv, Fig. 8. (Non *Unio ambiguus* Phil. 1847^26^), Iredale^43^, p. 190.

*Centralhyria angasi subjecta* Iredale, 1934^9^, p. 67 (in part), Iredale^43^, p. 190.

**Figure 3:**
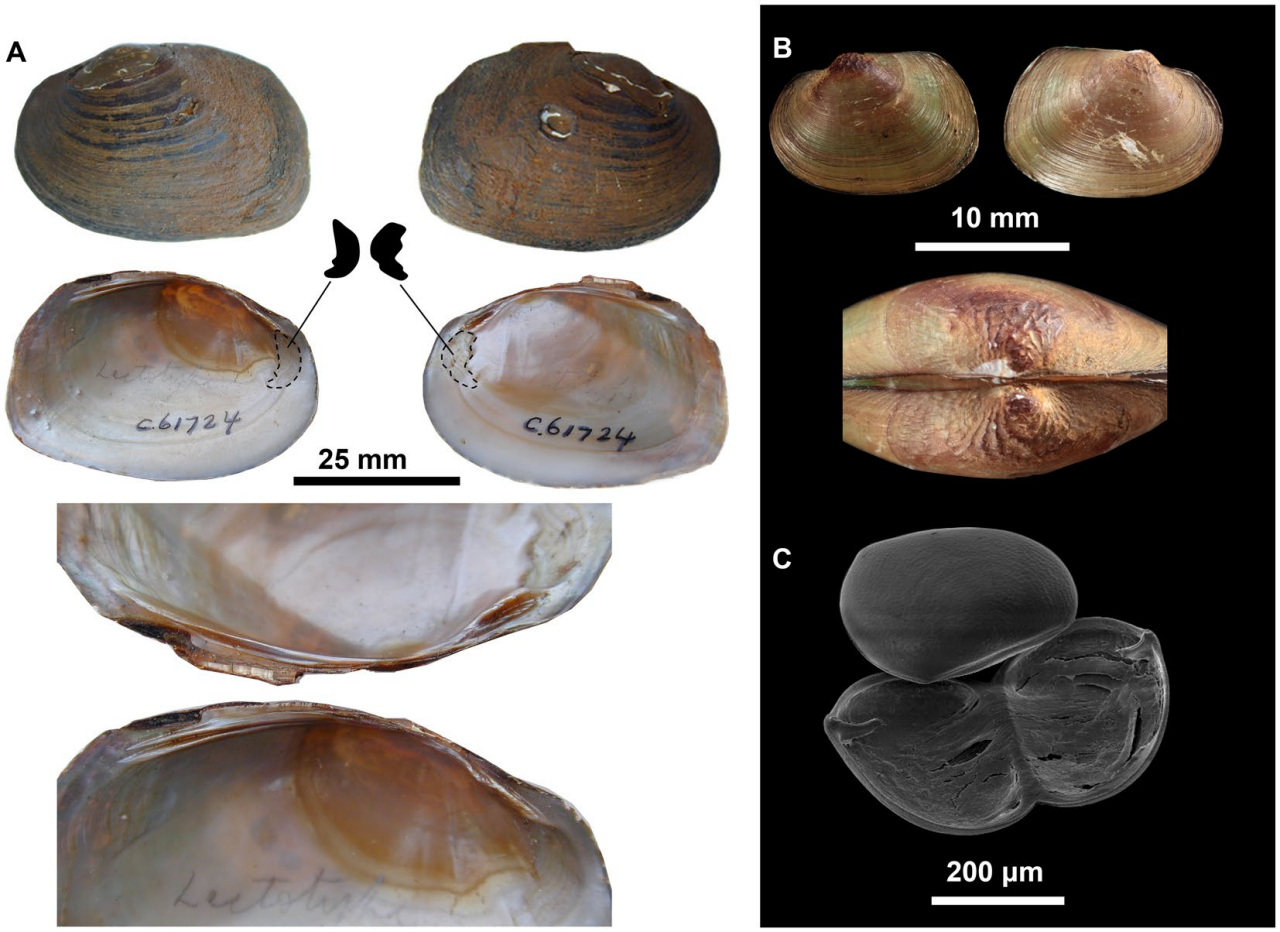
(**A**) *Westralunio ambiguus carteri* Iredale, 1934, Lectotype: Victoria Reservoir, Darling Range, 12 mi E of Perth, AMS C.061724. Detail of fusion in anterior muscle scars from either valve represented by dashed lines and black polygons. Bottom image showing detail of hinge teeth. Photos provided with permission by Dr Mandy Reid, AMS. (**B**) Valves and detail of sculptured umbo of a juvenile *W. carteri* from Yule Brook, Western Australia, UMZC 2013.2.9. Photo by Dr Michael W. Klunzinger. (**C**) Glochidia of *W. carteri* from Canning River, Western Australia. Photo by Dr Michael W. Klunzinger.

**Figure 4:**
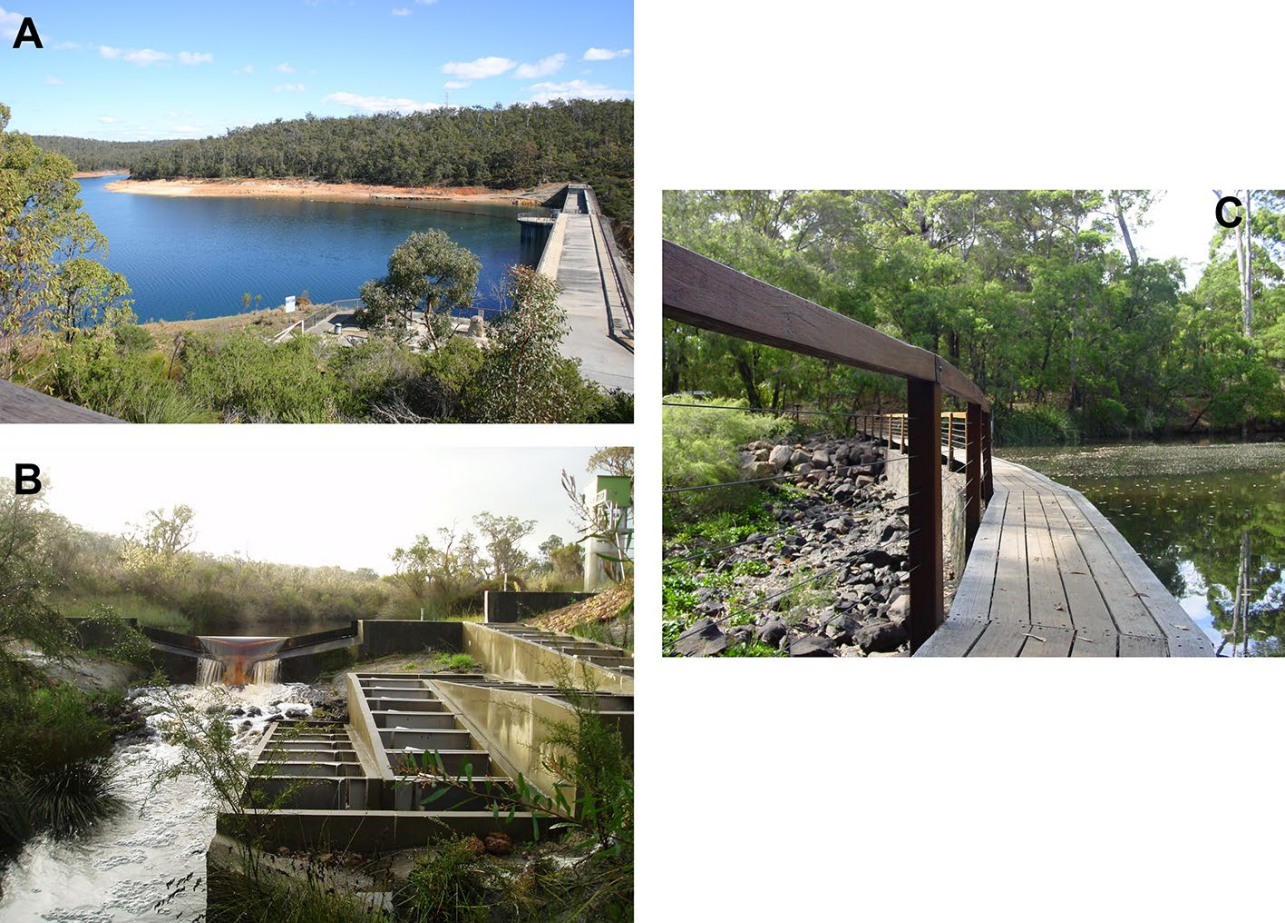
(**A**) Victoria Reservoir, Canning River, near Perth, Western Australia, type locality for *W. carteri*. Photo by Corey Whisson. (**B**) Goodga River, Western Australia, type locality for *W. inbisi inbisi*, at vertical slot fishway where holotype of *W. inbisi inbisi* was collected from. Photo provided with permission by Dr Stephen J. Beatty. (**C**) Margaret River, Western Australia, type locality for *W. inbisi meridiemus*, at Apex Weir. Photo by Dr Michael W. Klunzinger.

**Figure 5:**
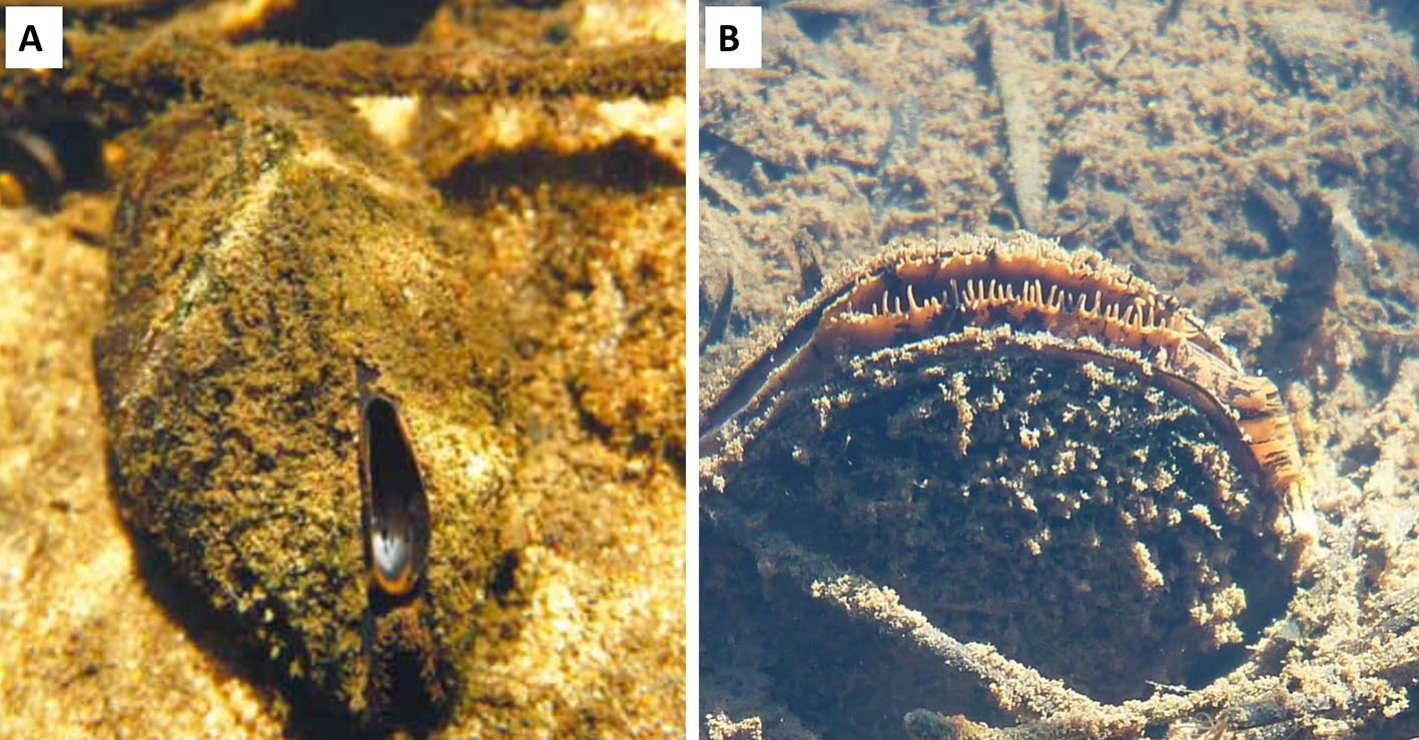
Live specimens of actively filtering freshwater mussels in the burrowed position. (**A**) *Westralunio carteri* (Iredale, 1934), Canning River at Kelmscott, Western Australia, inhalant siphon with 2–4 rows of papillae oriented toward substrate. Photo by Dr Michael W. Klunzinger. (**B**) *Westralunio inbisi meridiemus* subsp. nov., Canebreak Pool, Margaret River, Western Australia; inhalant siphon edges lined with protruding papillae facing towards water surface, away from substrate. Photo by Dr Michael W. Klunzinger.

**Figure 6:**
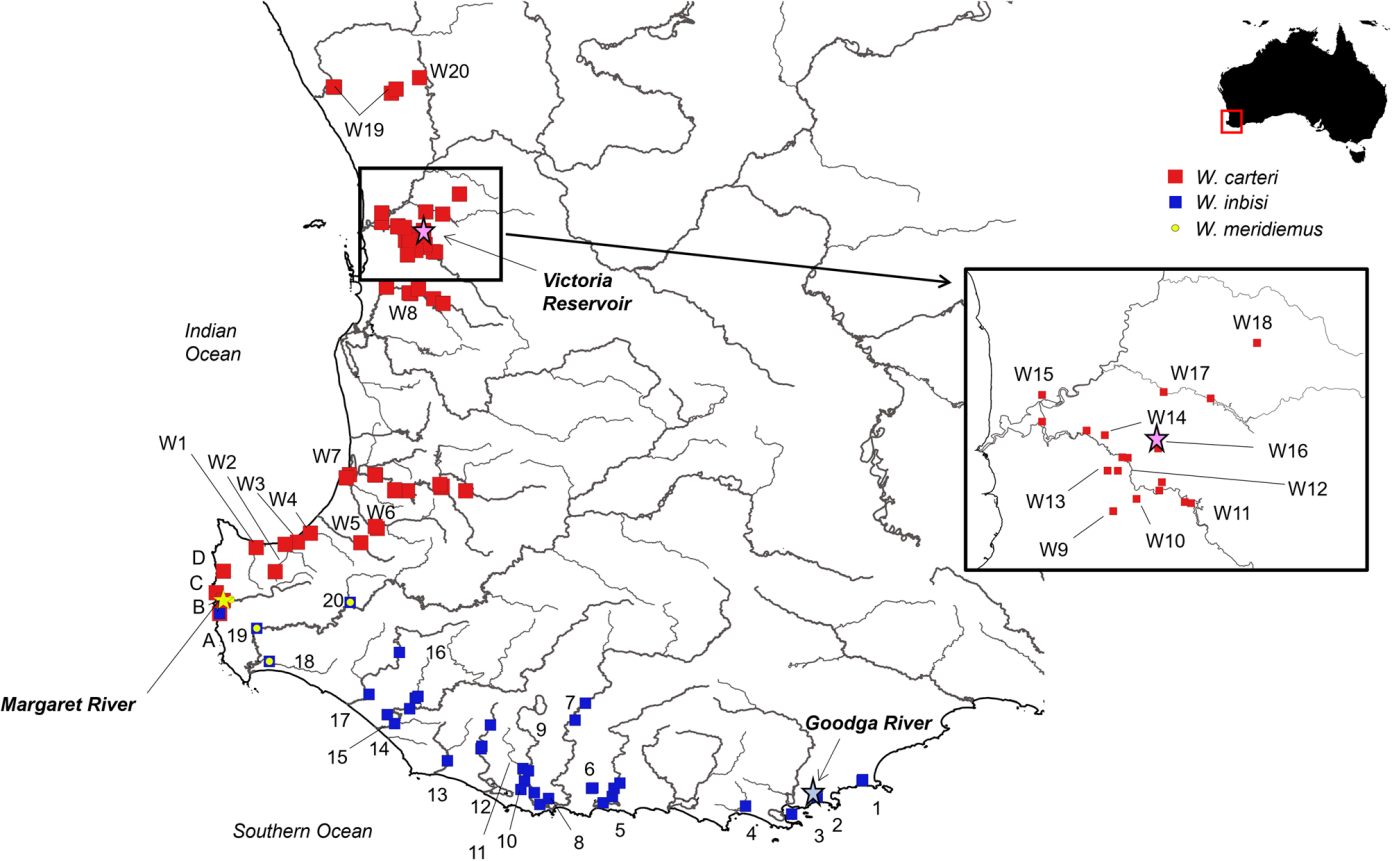
Distribution of the *Westralunio* specimens used for analyses in this study. Stars indicate type localities, labelled in bold, with colours corresponding to taxa (red—*W. carteri*, blue—*W. inbisi inbisi*, yellow—*W. inbisi meridiemus*). Waterbodies: South Coast: 1—Waychinicup R, 2—Goodga R, 3—King George Sound (N.B. museum records provided locality which we presume include freshwater streams or rivers draining to King George Sound rather than being collected from the marine environment), 4—Marbellup Bk, 5—Kent R, 6—Bow R, 7—Frankland R, 8—Walpole R, 9—Deep R, 10—Inlet R, 11—Weld R, 12—Shannon R, 13—Gardner R, 14—Warren R, 15—Lk Yeagarup, 16—Lefroy Bk, 17—Donnelly R, 18—Scott R, 19—Chapman Bk, 20—St. John Bk; Capes: A—Boodjidup Bk, B—Ellens Bk, C—Margaret R, D—Wilyabrup Bk; West Coast: W1—Carbunup R, W2—Vasse R, W3—Abba R, W4—Ludlow R, W5—Capel R, W6—Preston R, W7—Collie R, W8—Serpentine Res/R/Birrega Drain, W9—Wungong Bk, W10—Neerigen Bk, W11—Canning Res, W12—Canning R, W13—Southern R, W14—Yule Bk, W15—Swan R, W16—Victoria Res, W17—Helena R, W18—Lk Leschenaultia, W19—Gingin Bk, W20—Marbling Bk. Mapping methods provided in text. River basins within the South West Coast Drainage Division of Australia as defined under AWRC^102^. Spatial data were mapped as vector data in QGIS Desktop 3.24.3 (https://qgis.org/en/site/) using the GCS_GDA_1994 coordinate system^103^. The country outline for Australia was drawn from the GADM database (www.gadm.org), version 2.0, December 2011 under license. The rivers were mapped from the Linear (Hierarchy) Hydrography of Western Australia dataset (https://catalogue.data.wa.gov.au/dataset/hydrography-linear-hierarchy/resource/9908c7d1-7160-4cfa-884d-c5f631185859), under license.

**Figure 7:**
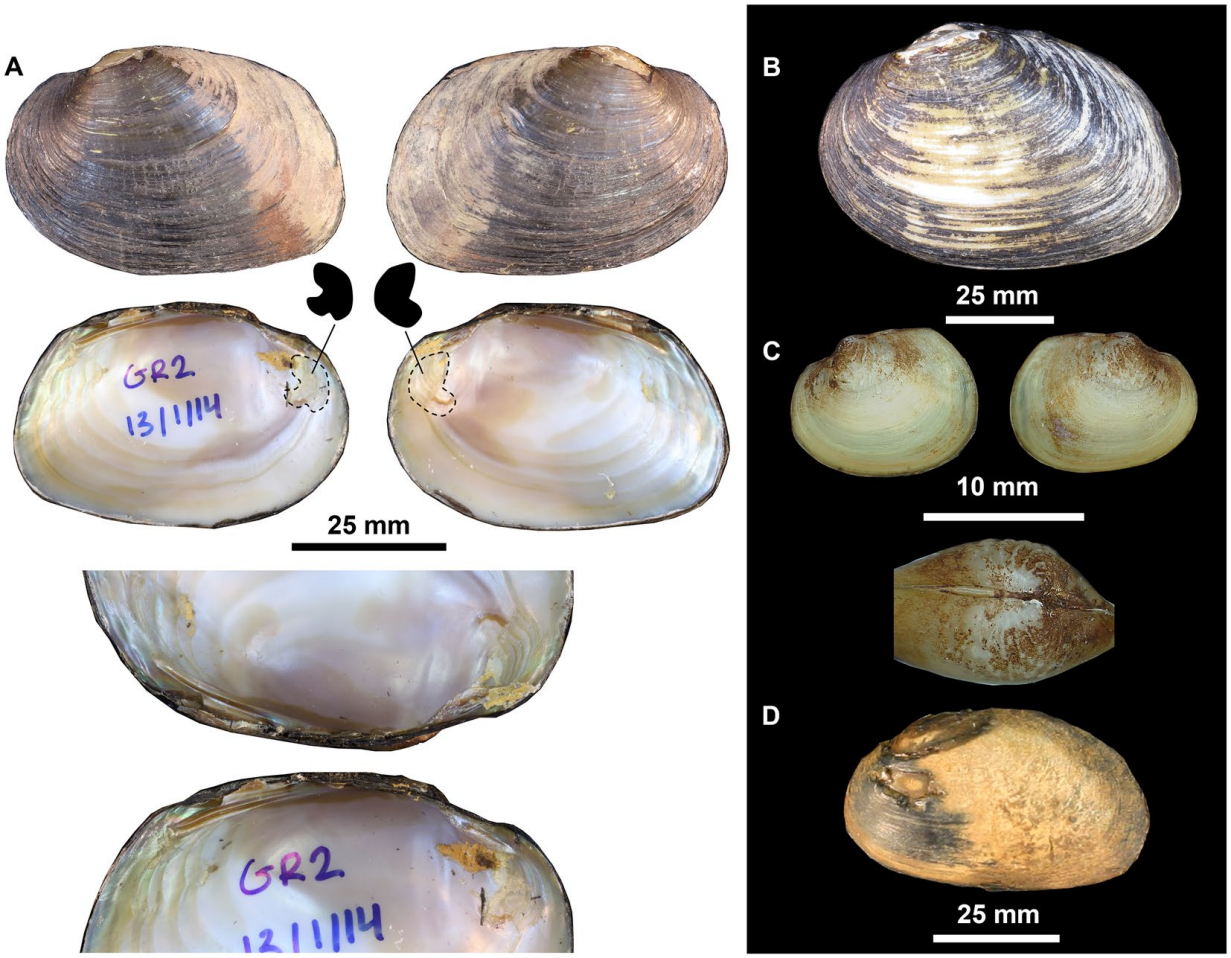
*Westralunio inbisi inbisi* subsp. nov., (**A**) Paratype: Goodga River, Western Australia, WAM S5620. Detail of fusion in anterior muscle scars from either valve represented by dashed lines and black polygons. Bottom image showing detail of hinge teeth. Photos by Corey Whisson. (**B**) Holotype: Goodga River, Western Australia, WAM S82756. Photo by Corey Whisson. (**C**) Valves and detail of sculptured umbo of a juvenile, Lake Yeagarup, Western Australia, WAM S82697. Photo by Dr Michael W. Klunzinger. (**D**) *Westralunio inbisi meridiemus* subsp. nov. Holotype: Margaret River, Western Australia, WAM S56235. Photo by Corey Whisson.

*Westralunio carteri* Iredale^9^: McMichael & Hiscock^10^pl. viii, Figs. 1, 2, 3, 4, 5, 6 and 7, pl. xvii, Figs. 4, 5.

#### Type material

**Lectotype**: AMS C.61724 (Fig. 3A) *Westralunio ambiguus carteri* Iredale, 1934^9^.

**Paralectotypes:** AMS C.170635 *Westralunio ambiguus carteri* Iredale, 1934^9^ (*n* = 4).

**Type locality**: Victoria Reservoir, Darling Range, 12 miles east of Perth, Western Australia (Fig. 4A).

**Lectotype:** BMNH 1840–10-21–29 *Centralhyria angasi subjecta* Iredale (selected by McMichael & Hiscock^10^).

**Type locality:** Avon River, Western Australia.

**Material examined for redescription:** For *W. carteri* (= “*W. carteri*” I), molecular data examined included 52 and 61 individual COI mtDNA and 16S rDNA sequences, respectively, for species delimitation. Additionally, Fourier shell shape outline analysis and traditional shell morphometric measurements were examined from 238 and 290 individuals, respectively. Complete details on all specimens examined are provided in Supplementary Table S1.

**ZooBank registration:** urn:lsid:zoobank.org:act:6B740F4D-40C3-4D6A-8938-B0FD7FD1F6D7.

**Etymology:** The species name *carteri* is most likely named after the surname of the collector who provided original type specimens to the Australian Museum, although Iredale^9^ did not specify this as the case. We have applied ICZN Articles 46.1 and 47.1^44^, designating *W. carteri* as the nominotypical species.

**Revised diagnosis:** Specimens of *W. carteri* are distinguished from other Australian *Westralunio* taxa by having shell series that are significantly larger and more elongated than *Westralunio inbisi inbisi* subsp. nov., but not different from *Westralunio inbisi meridiemus* subsp. nov. The species has 10 diagnostic nucleotides at COI (57 G, 117 T, 210 G, 249 T, 255 C, 345 G, 423 T, 447 T, 465 A, 499 T) and 13 at 16S (137 T, 155 C, 228 C, 229 T, 260 G, 290 A, 305 G, 307 T, 310 A, 311 C, 321 T, 330 A, 460 A), which differentiate it from its sister taxa, *W. inbisi inbisi* and *W. inbisi meridiemus* (each described below) using ASAP and TCS species delimitation models.

#### Redescription

This species is of the ESU “*W. carteri*” I^27,28^.

**Shell morphology:** Shells of relatively small to medium size, generally less than 70 mm in length, but to a maximum length of approximately 100 mm^10,45^, MHI 46–89%; anterior portion of shell with moderate development, BLI 22–49%; larger shells with abraded umbos scarcely winged; wing development variable, generally decreasing with size, BHI 76–104% (Table 2). Shell outline oblong-ovate to rounded; posterior end obliquely to squarely truncate, anterior end round; ventral edge slightly curved, nearly straight in larger specimens; hinge line curved, hinge strong. Umbos usually abraded in specimens > 20 mm in length; unabraded umbos with distinctive v- or w-shaped plicated sculpturing (Fig. 3B and Zieritz et al.^46^). Shell substance typically thick; shells of medium width with pronounced posterior ridge; periostracum smooth, dark brown to reddish, with fine growth lines. Pallial line less developed in smaller specimens and prominent only in large specimens (e.g., > 60 mm TL). Lateral teeth longer and blade-like, slightly serrated to smooth and singular in left valve, fitting into deep groove in right valve; pseudocardinal tooth in right valve coarsely serrated, thick, and erect, fitting into deeply grooved socket in left valve. Anterior muscle scars well impressed and anchored deeply in larger specimens; anterior retractor pedis and protractor pedis scars both small and fused with adductor muscle scar; posterior muscle scars lightly impressed; dorsal muscle scars usually with two or three deep pits anchored to internal umbo region.

**Anatomy:** Supra-anal opening absent, siphons of moderate size, not prominent but protrude beyond shell margin in actively filtering live specimens, pigmented dark brown with mottled lighter brown to orange splotches; inhalant siphon aperture about 1.5 times size of exhalant and bearing 2–4 rows of internal papillae (Fig. 5A); ctenidial diaphragm relatively long and perforated. Outer lamellae of outer ctenidia completely fused to mantle, inner lamellae of inner ctenidia fused to visceral mass then united to form diaphragm; palps relatively small, usually semilunar in shape; marsupium well developed as a distinctive swollen interlamellar space in the middle third of the inner ctenidium of females. Outer ctenidia in both sexes thin, with numerous, short intrafilamentary junctions and few, irregular interlamellar junctions; in females similar, but marsupium has numerous, tightly packed, well-developed interlamellar junctions. Thus, brooding in females is endobranchous.

**Life history:** Sexes are separate in *W. carteri,* and hermaphroditism appears to be rare^47–49^. Males and females both produce gametes year-round but brooding of glochidia appears to be seasonal and ‘tachyticitc’ (i.e., as defined by Bauer & Wächtler^19^, fertilisation and embryonic development occurring in late winter/early spring and glochidia release in early summer)^50^. Glochidia are released within vitelline membranes, embedded in mucus which extrude from exhalant siphons of females (i.e., 'amorphous mucus conglutinates') during spring/summer. Glochidia attach to host fishes and live parasitically on fins, gills or body surfaces for 3–4 weeks while undergoing metamorphosis to the juvenile stage. Host fishes which have been shown to support glochidia metamorphosis to the juvenile stage in the laboratory include *Afurcagobius suppositus* (Sauvage, 1880^51^), *Gambusia holbrooki* (Girard, 1859^52^), *Nannoperca vitttata* (Castelnau, 1873^53^), *Pseudogobius olorum* (Sauvage, 1880^51^) and *Tandanus bostocki* Whitley, 1944^54^ but not *Carassisus auratus* Linnaeus, 1758^31^ or *Geophagus brasiliensis* (Quoy & Gaimard, 1824^55^)^47^. Wild-caught fishes observed to be carrying *W. carteri* glochidia have included *A. suppositus*, *Bostockia porosa* Castelnau, 1873^53^, *G. holbrooki*, *Galaxias occidentalis* Ogilby, 1899^56^, *N. vittata*, *P. olorum*, *T. bostocki*, *Leptatherina wallacei* (Prince, Ivantsoff & Potter, 1982^57^), and *Phalloceros caudimaculatus* (Hensel, 1868^58^)^47^. Juveniles which have detached from host fishes have a characteristic ciliated foot and two distinct adductor muscles^47^. Probable age at maturity is 4–6 years old and estimated longevity is at least 36 to 52 years^59^. Inheritance of mitochondria is doubly uniparental^60^.

**Glochidium:** Following release, glochidia hatch from vitelline membranes but remain tethered by a larval thread and characteristically ‘wink’; valves with single adductor muscle; shells subtriangular and scalene in shape with smooth surface which lack surface spikes and dotted with pores, 305–310 μm long, 249–253 μm high and have a hinge length of 210–214 μm; apex of the ventral edge protrudes and is off-centre and closest to the posterior region of the glochidial shell, giving a sub-triangular scalene shape; larval teeth slightly curved towards adductor muscle with concave protuberance on base of the right valve tooth and convex protuberance on base of the left valve tooth; larval tooth of the right valve lanceolate, terminating with three sharp cusps; tooth of left valve blunt with two rounded cusps and groove at the midpoint to accommodate the middle cusp of the right valve; larval teeth lack microstylets (Fig. 3C and Klunzinger et al*.*^48^).

**Distribution:** Found in freshwater catchments from Gingin Brook, north of Perth to westerly flowing drainages north and west of the Blackwood River, within 150 km of the coast^28,61^ (Fig. 6).

**Habitat:** Found in freshwater streams, rivers and sometimes lakes or wetlands with permanent water, salinities less than about 3.0 mg/L, pH ranging from about 4.5 to 10 and more common in habitats not prone to nutrient pollution^61^.

**Comments:** McMichael & Hiscock^10^ suggested that the species aligns with other Velesunioninae in having smooth umbos, later refuted by Zieritz et al*.*^46^, as illustrated in Fig. 3B. Additionally, Iredale^9^ separated the genus *Westralunio* from *Velesunio* based on adult hinge tooth morphology, such that pseudocardinal hinge teeth are erect, serrated and strongly grooved in *Westralunio* as opposed to *Velesunio* which are suggested as not serrated and not strongly grooved. We contend that while *W. carteri* typically does have serrated pseudocardinal teeth that are usually erect/conspicuous and strongly grooved, so too are some *Velesunio* specimens (M. Klunzinger, unpublished data). In terms of distribution, there is one record of a specimen from the Gascoyne River collected ca. 1891 (BMNH-MP-110 listed as *Diplodon ambiguus* Parreyss in Philippi^26^ = *Unio philippianus* Küster, 1861^62^; from Graf & Cummings^63^) which is well north of the species currently known range boundary. It is unclear whether the species occurs in that river as it has not been collected from north of the Moore-Hill Basin apart from this individual record.

### *Westralunio flyensis* (Tapparone Canefri, 1883)

#### Synonymy

*Unio (Bariosta) flyensis* Tapparone Canefri, 1883^24^, pp. 293–294, text Fig. 1.

*Diplodon (Hyridella) flyensis* (Tapp. Can.^24^), Simpson^64^, p. 1295.

*Hyridella flyensis* (Tapp. Can.^24^), Haas^65^, pp. 74–75, pl. ii, Figs. 4 and 5.

*Westralunio flyensis* (Tapp. Can.^24^), McMichael^66^, p. 41.

#### Type material

**Holotype:** The Holotype is held at Museo Civico di Storia Naturale, Genoa, Italy.

**Paratypes:** Two Paratypes are held at Museo Civico di Storia Naturale, Genoa, Italy.

**Type Locality:** Fly River, Papua New Guinea.

**Description:** As described by McMichael & Hiscock^10^.

**Distribution:** Southern rivers of New Guinea.

### *Westralunio albertisi* Clench, 1957

#### Type material

**Holotype:** MCZ 212908.

**Paratype:** AMS C.62268.

**Type Locality:** inland from Daru, Papua.

**Paratype:** MCZ 191391, Lake Murray, Fly River, Papua New Guinea.

**Description:** As described by McMichael & Hiscock^10^.

**Distribution:** Lakes of the Fly River district, Papua New Guinea.

### *Westralunio inbisi* sp. nov.

#### *Westralunio inbisi inbisi* subsp. nov.

##### Type material

**Holotype:** WAM S82756 (Fig. 7A–C), collected by M.W. Klunzinger.

**Type locality:** Goodga River at vertical slot fishway, Western Australia (34.9485°S, 118.0799°E, GDA94) (see Fig. 4B).

**Paratypes:** WAM S56200, WAM S56201, WAM S56202, WAM S56203, collected by M. W. Klunzinger.

**Type locality:** Goodga River, Western Australia (34.9597°S, 118.0981°E, GDA94).

**Material examined:** For *W. inbisi inbisi* (= “*W. carteri*” II), molecular data examined included 82 and 93 individual 16S rDNA and COI mtDNA sequences, respectively, for species delimitation. Additionally, Fourier shell shape outline analysis and traditional shell morphometric measurements were examined from 127 and 139 individuals, respectively. Complete details on all specimens examined are provided in Supplementary Table S2.

**Etymology:** The specific epithet, *inbisi*, is derived from the Nyoongar word ‘inbi’, translating to ‘mussel, fresh water’ in English^67^.

**Diagnosis:** Specimens of *W. inbisi inbisi* are distinguished from other Australian *Westralunio* taxa by having shell series that are significantly smaller and less elongated than *W. carteri*, but not *W. inbisi meridiemus*. The subspecies has three diagnostic nucleotides at COI (75 A, 87 T, 318 T) and none at 16S, which differentiate it from its sister taxa, *W. carteri* and *W. inbisi meridiemus* using ASAP and TCS species delimitation models.

**Description:** This subspecies is of the ESU “*W. carteri*” II^27,28^. Shell morphology in juveniles and adults same as *W. carteri* as described above (see Fig. 7A–C). Total adult shell length generally < 80 mm but known to reach in excess of 90 mm^68,69^. MHI 55–74%; anterior portion of shell with moderate development, BLI 23–51%; larger shells with abraded umbos scarcely winged; wing development variable, generally decreasing with size, BHI 79–99% (Table 2); anatomy same as *W. carteri*; life history: sexes appear to be separate based on macroscopic examinations of marsupia in non-gravid and gravid females, examined in the field in September and March 2011, respectively; wild-caught fishes from Fly Brook, Lefroy Brook and Shannon River, observed to be carrying what we presume to be *W. inbisi inbisi* glochidia have included *B. porosa*, *N. vittata* and *T. bostocki*^47^. Mature glochidia of *W. inbisi inbisi* have not been formally described. Reproductive phenology, age and growth have not been elucidated in *W. inbisi inbisi*.

**Distribution:** Found in southerly to south-westerly flowing freshwater streams, rivers and sometimes lakes or wetlands with water salinities less than approximately 3.0 mg/L from Boodjidup Brook in the Capes region to the west of the Blackwood River catchment to Waychinicup River within 150 km of the coast, primarily along the South Coast of Western Australia^27–29,61,69^ (Fig. 6).

**Habitat:** Similar to *W. carteri* although habitats can also include perched dune lakes. Waters that *W. inbisi inbisi* inhabit are often more tannin stained due to their occurrence in more heavily forested catchments with greater densities of native riparian vegetation than for *W. carteri*.

### *Westralunio inbisi meridiemus* subsp. nov

#### Type material

**Holotype:** WAM S56235 (Fig. 7D), collected by M.W. Klunzinger.

**Paratypes:** WAM S56236, WAM S56237, WAM S56238, WAM S56239, collected by M. W. Klunzinger.

**Type locality:** Apex Weir, Margaret River, Western Australia (33.942995°S, 115.073151°E, GDA94) (see Fig. 4C).

**Material examined:** For *W. inbisi meridiemus* (= “*W. carteri*” III), molecular data examined included 9 and 12 individual 16S rDNA and COI mtDNA sequences, respectively, for species delimitation. Additionally, Fourier shell shape outline analysis and traditional shell morphometric measurements were examined from 12 individuals each. Complete details on all specimens examined are provided in Supplementary Table S3.

**Etymology:** The subspecific epithet, *meridiemus*, is derived from Latin ‘meridiem’, translating to ‘southwest’ in English in reference to the location of its type locality, which sits in the southwestern region of Western Australia.

**Diagnosis:** Specimens of *W. inbisi meridiemus* have five diagnostic nucleotides at COI (69 C, 123 C, 126 T, 483 A, 526 A) and none at 16S, which differentiate it from its sister taxa, *W. carteri* and *W. inbisi inbisi* using ASAP and TCS species delimitation models.

**Description:** This subspecies is of the ESU “*W. carteri*” III. Shell morphology in juveniles unknown, but adults same as *W. inbisi inbisi* and *W. carteri* as described above; total adult shell length generally < 80 mm. MHI 60–71%; anterior portion of shell with moderate development, BLI 28–35%; larger shells with abraded umbos scarcely winged; wing development variable, generally decreasing with size, BHI 84–93% (Table 2); anatomy same as *W. carteri* and *W. inbisi inbisi*, including siphon pigmentation and morphology (illustrated in Fig. 5B). Life history observations for this ESU cannot be derived from existing field observations as all known populations overlap the distribution of either *W. carteri* (in Margaret River) or *W. inbisi inbisi* (in the lower Blackwood Basin); however, mussels from those locations appear to have separate sexes based on macroscopic examinations of marsupia in both gravid and non-gravid females. Similarly, gravid female mussels have been observed from Margaret River during late spring to summer (November to December). Species of wild-caught fishes from Canebreak Pool in Margaret River that have been observed carrying glochidia include *G. occidentalis* and *N. vittata*^47^. Reproductive phenology, age and growth have not been elucidated in *W. inbisi meridiemus*.

**Distribution:** Found in the neighbouring catchments of Margaret River and the Blackwood River of Western Australia, where it is sympatric with *W. carteri* and *W. inbisi inbisi*, respectively^27,28^ (Fig. 6).

**Habitat:** Similar to *W. carteri* and *W. inbisi inbisi* in either lotic or lentic freshwater rivers, streams, and pools with varying degrees of riparian vegetation.

## Discussion

This study is the first to integrate molecular species delimitation and morphological analyses to describe new taxa of Australian freshwater mussels. In their review of the taxonomy, phylogeography and conservation of freshwater mussels in Australasia, Walker et al.^14^ highlighted the need for such a taxonomic framework that uses both genetic and morphological data to gain a better understanding of species delimitation within this group. Overall, the study illustrates the value of using multiple data sources for species delimitation for cryptic taxa, provides an example when it is appropriate to recognise subspecies, and describes a case study of an IUCN listed species that would have to be re-assessed in terms of conservation status following the application of a robust taxonomic framework for recognising species boundaries.

In this study, we aimed to use multiple lines of evidence to investigate whether the three ESUs previously identified for the freshwater mussel *Westralunio carteri*^27,28^ should be recognised as separate species. Using three species delimitation models run for 164 COI mtDNA sequences, a combination of both traditional indices of shell morphology and Fourier shell shape analyses and geographical distribution records, our results provided a clear case for the recognition of at least two separate species—*Westralunio carteri* (Iredale, 1934^9^) which is found in rivers draining the western coast, and *W. inbisi* sp. nov. which occurs in rivers draining the southern and lower southwestern coast of southwestern Australia. The recognition of these two separate species was well supported, with congruent data sets confirming that they are both morphologically and genetically divergent.

Carstens et al.^70^ have argued strongly that species should be delimited based on the congruence of multiple data sets that could include genetic, morphological and distributional data, as was done in this study. This is particularly true for cryptic taxa. The use of an integrated approach for resolving taxonomic uncertainties for freshwater mussels has growing support. For example, Johnson et al.^4^ used multiple lines of evidence to show that current taxonomy overestimated species diversity within the imperilled freshwater mussel genus *Cyclonaias* in North America. Similarly, Morrison et al.^71^ tested species boundaries in the North American *Pleurobema* species complex using genetic and morphological data, finding that the most likely scenario was that the two named species they investigated were members of a single, widespread species. Despite this growing trend of using multiple sources of evidence for species delimitation, several freshwater mussel taxa have been named based on shell morphology alone in recent decades. For example, the hyriids *Lortiella opertanea* Ponder & Bayer, 2004^11^ and *Triplodon chodo* Mansur & Pimpão, 2008^72^ were erected as new species based on shell shape indices and sculpture pattern, respectively. Molecular analysis has yet to corroborate the taxonomy in these and other recently described freshwater mussels. The propensity of shell shape to vary with the environment within freshwater mussel species can render it an often-unreliable character on which to base species taxonomy^12,14,73,74^. Alternatively, relying on genetic data without morphological support can also be problematic; however new species have been raised using this method^2,3^. While this may not be appropriate for practical purposes^75,76^, there is no doubt that recognising genetic differences between populations is an important aspect of describing biodiversity and indeed, has been used in several species’ concepts^77,78^.

Our study also provides an example where subspecies have been described in recognition of the existence of phenotypically similar, but genetically distinct evolutionary lineages within the *W. carteri* species complex. In our case, shell morphology for *W. inbisi inbisi* and *W. inbisi meridiemus* was mostly similar, yet these taxa possessed COI character attributes that were unique to each, largely geographically separated lineage, suggesting a degree of reproductive isolation and an evolutionarily significant process. We did have a low replicate number of shells of *W. inbisi meridiemus* to examine which may have accounted for the lack of statistical differences between this subspecies and *W. inbisi inbisi* if indeed differences might exist. While there is additional material available from Margaret and Blackwood Rivers in museum collections, we were restricted to examining only shells from which genetic information is available given Benson et al.^28^ found both taxa in the two river basins. The definition of a subspecies can vary but is widely accepted as an aggregate of phenotypically similar populations of a species inhabiting a geographic subdivision of the range of that species and differing taxonomically from other populations of that species^79^. In their study of land snails, Páll-Gergely et al*.*^80^ suggest restricting subspecies to cryptic species delimitation, for example when molecular data support lineage separation but where no clear morphological differences are currently known. We agree with this definition but caution that molecular data can exhibit differentiation related to population structure and not speciation. Fixed molecular differences are critical to confirming reciprocal monophyly when molecular phylogenetic methods are employed. The use of subspecies in the Hyriidae is relatively common. Of the 261 names available for species or subspecies of Hyriidae, 57 (21.8%) have been used either exclusively as subspecies or as either species or subspecies^9,11,18,20,23,43,64,81–87^. Here we recognise subspecies based on congruence in genetic and geographical differentiation but chose not to raise “*W. carteri*” III to species level given there was no significant difference in shell morphology between it and “*W. carteri*” II and because not all molecular species delimitation models revealed a separate taxon for “*W. carteri*” III.

These results confirm that current taxonomy underestimates species diversity of freshwater mussels in southwestern Australia and that this has implications for the listing of *W. carteri* as a threatened species. Freshwater mussels are amongst the most threatened aquatic species worldwide, and many authors have expressed concern about the global decline of this group^88–90^. It is not surprising therefore that a recent review by Benson et al.^91^ revealed that 44% (18 of 41 species) of described freshwater mussel species known to occur in Mediterranean-climate regions have been listed globally as either Critically Endangered, Endangered or Vulnerable on the IUCN Red List. A further six species (15%) have been classified as Near Threatened. Freshwater mussels are also notorious in having shells which are morphologically plastic within the same taxa^12^ and taxa which have morphologically similar shells but are molecularly different, leading to ‘cryptic speciation’^92^. Given the increasing interest in using multiple data sets to confirm species boundaries in the group, amendments to the Red List conservation status of these listed species can be anticipated as species delimitation based originally on morphological characters is further clarified with this integrative taxonomy approach. In the case of *W. carteri*, the species is currently listed as ‘Vulnerable’ internationally (IUCN Red List), nationally under the Australian *Environmental Protection and Biodiversity Conservation Act 1999* (EPBC Act) and at the state level under the Western Australia *Biodiversity Conservation Act 2016*. More recently, in a preliminary analysis based on past distribution data^61^, Klunzinger et al.^27^ suggested that an estimated reduction of 72% in extent of occurrence (EOO) of the *W. carteri* lineage identified here might qualify this species as ‘Endangered’ under criterion A2c of the IUCN Red List. More robust analyses which include recent distribution records would be needed to confirm the conservation status of this species as well as the new species described in this study. Our evidence suggests that once formal re-diagnoses and descriptions of the *Westralunio* taxa are published, fresh nominations for listing as threatened would be required. This would entail submission of a nomination to the Threatened Species Scientific Committee to amend the conservation status of *W. carteri*, and the preparation of new nominations for one or both new subspecies if deemed necessary. As more studies use an integrative approach for delineating freshwater mussel species, implications for conservation are inevitable, and this is likely to take place on a global scale. For example, the suggestion that the freshwater mussel species *Pleurobema clava* (listed as Endangered under the Endangered Species Act in the USA) and *P. oviforme* (a species being considered for listing) are members of a single, widespread species will have management implications for these species^71^.

Several research gaps in our knowledge of *Westralunio* will benefit from future investigation. The position of *W. albertisi* and *W. flyensis* from West Papua and Papua New Guinea within the genus *Westralunio* is unclear^10,14^ and employing molecular analyses will undoubtedly resolve this biogeographic and taxonomic conundrum. At a higher classification level, given that both species of juvenile Australian *Westralunio* have distinctive v- or w-shaped shell sculpturing on their umbos^46^, in combination with strongly grooved and serrated pseudocardinal hinge teeth in adult shells, calls into question their placement within the Velesunioninae defined by McMichael & Hiscock^10^. This is corroborated by phylogenetic data presented by Graf et al*.*^21^ and Santos-Neto et al*.*^22^ who showed *Westralunio* separate to the other Velesunioninae. However, until complete data are available for other *Westralunio* and other velesunionine species, we are reluctant to make any changes to the current arrangement of the subfamily. Also of value would be an examination of glochidia morphology and morphometry. Glochidia of *W. carteri* were described by Klunzinger et al*.*^48^, but the morphology and morphometry of glochidia from *W. inbisi inbisi* and *W. inbisi meridiemus* are entirely unknown and worthy of investigation. Indeed, glochidia morphology and morphometry has been shown to have taxonomic value for other species^19^. For example, Jones et al*.*^93^ showed divergence in larval tooth arrangement and shell size in the south-east Australian *Hyridella australis* (Lamarck, 1819^39^), *Hyridella depressa* (Lamarck, 1819^39^) and *Cucumerunio novaehollandiae* (Gray, 1834^94^). Pimpão et al*.*^95^ were able to distinguish several species of Brazilian Hyriidae using glochidial characters and made taxonomic changes to some genera and subgenera on this basis. More recently, Melchior et al*.*^96^ presented contrasting glochidia release strategies and glochidia size in between two sympatric species of *Echyridella* from New Zealand, further strengthening the taxonomic division between *Echyridella menziesii* (Gray, 1843^97^) and *Echyridella aucklandica* (Gray, 1843^97^) recognised by Marshall et al*.*^20^.

This study has highlighted the utilisation of morphology and phylogeography to make sound decisions on drawing taxonomic boundaries between ESUs. Formalising the taxonomy of ESUs identified by Klunzinger et al*.*^27^ and Benson et al*.*^28^ for *Westralunio* taxa will be beneficial for conservation management of the species and subspecies identified in this study. We suggest that a similar approach of taxonomic division may be applied to other freshwater fauna of the region (and elsewhere), such as for the multiple lineages of pygmy perches or galaxiid fishes alluded to by Morgan et al.^98^ and Buckley et al*.*^99^. Furthermore, the taxonomic approach illustrated in our study should be applied to other as yet undescribed ‘cryptic species’ of Australian freshwater mussels^92,100,101^ as a way forward in resolving taxonomic uncertainty within the group.

## Methods

### Mapping and provenance

River basins within the South West Coast Drainage Division of Australia as defined under AWRC^102^. Spatial data were mapped as vector data in QGIS Desktop 3.24.3 (https://qgis.org/en/site/) using the GCS_GDA_1994 coordinate system^103^. The country outline for Australia was drawn from the GADM database (www.gadm.org), version 2.0, December 2011 under license. The rivers were mapped from the Linear (Hierarchy) Hydrography of Western Australia dataset (https://catalogue.data.wa.gov.au/dataset/hydrography-linear-hierarchy/resource/9908c7d1-7160-4cfa-884d-c5f631185859), under license.

### Gross anatomy

Siphon characters, which are not easily examined in preserved specimens (for example, due to tissue contraction and discolouration), were observed in live specimens of *W. carteri* in the Canning and Margaret Rivers with mask and snorkel and photographed in their natural state with a waterproof digital camera. Tissue anatomy of freshly dead and preserved specimens was examined employing well-established dissection methods^10^ on specimens collected for data published by Klunzinger et al*.*^27^. For shell sculpture examination, we drew on data published by Zieritz et al*.*^46^. Individual specimens of dry shells lacking soft anatomy, also examined for shell shape and measured morphometrically (see below) were examined for the arrangement of adductor and retractor muscle scars following McMichael & Hiscock^10^.

### Genetic methods

#### Sequence alignment construction

We assembled mitochondrial Cytochrome c Oxidase Subunit I (COI) and 16S rDNA (16S) gene sequences originally published by Klunzinger et al.^27^ and Benson et al.^28^. Available nuclear gene sequences (18S and 28S rDNA) were not used in our analysis because they have insufficient variation to be informative^28^. Sequences were retrieved from GenBank, along with sequences for two other Hyriid species for use as outgroups (Velesunioninae: *Velesunio ambiguous* (Philippi, 1847^26^) and Hyriinae: Hyridellini: *Cucumerunio novaehollandiae* (Gray, 1834^94^)) (see Table 3). We limited the outgroup species to two hyriids in our tree because of the extreme divergence of *W. carteri*, even from other Hyriidae (see Graf et al.^21^). Individual alignments for both genes were built using the ClustalW accessory application in Bioedit 7.2.5^104^, before inspecting and trimming to equal length in MEGA X version 10.1.8^105^.

**Table 3:** Taxa used for phylogenetic analyses.

BASIN/Locality	Waterbody	Taxon/ESU	COI	16S	Voucher/source
MANNING, NSW, Australia	Glochester River	*Cucumerunio novaehollandiae*	KP184901	KP184853	UMMZ 304501^21^
HAWKSBURY, NSW, Australia	Napean River	*Velesunio ambiguus*	KP184915	KP184868	FMNH 337195^21^
MOORE-HILL, WA, Australia	Gingin Brook	“*Westralunio carteri*” I	MT040666	–	WAM S82791^27^
SWAN COAST, WA, Australia	Lake Leschenaultia	“*Westralunio carteri*” I	MT040670 KP184918	MT040067 KP184871	WAM S82739^27^ UMMZ 304517^27^
SWAN COAST, WA, Australia	Marbling Brook	“*Westralunio carteri*” I	MT040671	–	WAM S82790^27^
SWAN COAST, WA, Australia	Neerigen Brook	“*Westralunio carteri*” I	KP184917	KP184870	UMMZ 304516^27^
SWAN COAST, WA, Australia	Wungong Brook	“*Westralunio carteri*” I	MT040656 MT040657 MT040658	– – -	WAM S56225^27^ WAM S56226^27^ WAM S56229^27^
MURRAY, WA, Australia	Serpentine River	“*Westralunio carteri*” I	MT040651 MT040652 MT040653 MT040654 MT040655 MT040664	– – – – – MT040065	WAM S56220^27^ WAM S56221^27^ WAM S56222^27^ WAM S56223^27^ WAM S56224^27^ WAM S82779^27^
COLLIE, WA, Australia	Collie River	“*Westralunio carteri*” I	MT040628 MT040629 MT040630 MT040631 MT040632 MT040665	– – – – – MT040066	WAM S56210^27^ WAM S56211^27^ WAM S56212^27^ WAM S56213^27^ WAM S56214^27^ WAM S82777^27^
PRESTON, WA, Australia	Preston River	“*Westralunio carteri*” I	MT040646 MT040647 MT040648 MT040649 MT040650	– – – – –	WAM S56215^27^ WAM S56216^27^ WAM S56217^27^ WAM S56218^27^ WAM S56219^27^
BUSSELTON COAST, WA, Australia	Capel River	“*Westralunio carteri*” I	MZ668727 MZ668728 MZ668729 MZ668730 MZ668731	MZ668847 MZ668848 MZ668849 MZ668850 MZ668851	WAM S112705^28^ WAM S112706^28^ WAM S112707^28^ WAM S112708^28^ WAM S112709^28^
BUSSELTON COAST, WA, Australia	Ludlow River	“*Westralunio carteri*” I	MZ668732 MZ668733 MZ668734 MZ668735 MZ668736	MZ668852 MZ668853 MZ668854 MZ668855 MZ668856	WAM S112710^28^ WAM S112711^28^ WAM S112712^28^ WAM S112713^28^ WAM S112714^28^
BUSSELTON COAST, WA, Australia	Abba River	“*Westralunio carteri*” I	MZ668737 MZ668738 MZ668739 MZ668740 MZ668741	MZ668857 MZ668858 MZ668859 MZ668860 MZ668861	WAM S112715^28^ WAM S112716^28^ WAM S112717^28^ WAM S112718^28^ WAM S112719^28^
BUSSELTON COAST, WA, Australia	Carbanup River	“*Westralunio carteri*” I	MZ668742 MZ668743 MZ668744 MZ668745 MZ668746	MZ668862 MZ668863 MZ668864 MZ668865 MZ668866	WAM S112720^28^ WAM S112721^28^ WAM S112722^28^ WAM S112723^28^ WAM S112724^28^
BUSSELTON COAST, WA, Australia	Ellens Brook	“*Westralunio carteri*” I	MZ668752 MZ668753 MZ668754 MZ668755 MZ668756	MZ668872 MZ668873 MZ668874 MZ668875 MZ668876	WAM S112730^28^ WAM S112731^28^ WAM S112732^28^ WAM S112733^28^ WAM S112734^28^
BUSSELTON COAST, WA, Australia	Wilyabrup Brook	“*Westralunio carteri*” I	MZ668747 MZ668748 MZ668749 MZ668750 MZ668751	MZ668867 MZ668868 MZ668869 MZ668870 MZ668871	WAM S112725^28^ WAM S112726^28^ WAM S112727^28^ WAM S112728^28^ WAM S112729^28^
BUSSELTON COAST, WA, Australia	Boodjidup Brook	“*Westralunio carteri*” I “ “ “ “*Westralunio carteri*” II	MZ668812 MZ668813 MZ668814 MZ668815 MZ668816	MZ668932 MZ668933 MZ668934 MZ668935 MZ668936	WAM S112790^28^ WAM S112791^28^ WAM S112792^28^ WAM S112793^28^ WAM S112794^28^
BUSSELTON COAST, WA, Australia	Margaret River	“*Westralunio carteri*” III “ “ “ “ “*Westralunio carteri*” I “*Westralunio carteri*” III “*Westralunio carteri*” I “*Westralunio carteri*” III “	MT040641 MT040642 MT040643 MT040644 MT040645 MZ668757 MZ668758 MZ668759 MZ668760 MZ668761	MT040060 MT040061 – – – MZ668877 MZ668878 MZ668879 MZ668880 MZ668881	WAM S56235^27^ WAM S56236^27^ WAM S56237^27^ WAM S56238^27^ WAM S56239^27^ WAM S112735^28^ WAM S112736^28^ WAM S112737^28^ WAM S112738^28^ WAM S112739^28^
BLACKWOOD, WA, Australia	Scott River	“*Westralunio carteri*” II “ “ “*Westralunio carteri*” III “*Westralunio carteri*” II	MZ668762 MZ668763 MZ668764 MZ668765 MZ668766	MZ668882 MZ668883 MZ668884 MZ668885 MZ668886	WAM S112740^28^ WAM S112741^28^ WAM S112742^28^ WAM S112743^28^ WAM S112744^28^
BLACKWOOD, WA, Australia	Chapman River	“*Westralunio carteri*” III “*Westralunio carteri*” II “ “ “*Westralunio carteri*” III	MZ668807 MZ668808 MZ668809 MZ668810 MZ668811	MZ668927 MZ668928 MZ668929 MZ668930 MZ668931	WAM S112785^28^ WAM S112786^28^ WAM S112787^28^ WAM S112788^28^ WAM S112789^28^
BLACKWOOD, WA, Australia	St. Johns Brook	“*Westralunio carteri*” II “ “ “ “ “ “*Westralunio carteri*” III “*Westralunio carteri*” II	MT040659 MT040660 MT040661 MZ668832 MZ668833 MZ668834 MZ668835 MZ668836	– MT040662 – MZ668952 MZ668953 MZ668954 MZ668955 MZ668956	WAM S82773^27^ WAM S66164^27^ WAM S66165^27^ WAM S112810^28^ WAM S112811^28^ WAM S112812^28^ WAM S112813^28^ WAM S112814^28^
DONNELLY, WA, Australia	Donnelly River	“*Westralunio carteri*” II	MZ668802 MZ668803 MZ668804 MZ668805 MZ668806	MZ668922 MZ668923 MZ668924 MZ668925 MZ668926	WAM S112780^28^ WAM S112781^28^ WAM S112782^28^ WAM S112783^28^ WAM S112784^28^
WARREN, WA, Australia	Lake Yeagarup	“*Westralunio carteri*” II	MZ668797 MZ668798 MZ668799 MZ668800 MZ668801	MZ668917 MZ668918 MZ668919 MZ668920 MZ668921	WAM S112775^28^ WAM S112776^28^ WAM S112777^28^ WAM S112778^28^ WAM S112779^28^
WARREN, WA, Australia	Warren River	“*Westralunio carteri*” II	MZ668767 MZ668768 MZ668769 MZ668770 MZ668771	MZ668887 MZ668888 MZ668889 MZ668890 MZ668891	WAM S112745^28^ WAM S112746^28^ WAM S112747^28^ WAM S112748^28^ WAM S112749^28^
SHANNON, WA, Australia	Gardner River	“*Westralunio carteri*” II	MZ668772 MZ668773 MZ668774 MZ668775 MZ668776	MZ668892 MZ668893 MZ668894 MZ668895 MZ668896	WAM S112750^28^ WAM S112751^28^ WAM S112752^28^ WAM S112753^28^ WAM S112754^28^
SHANNON, WA, Australia	Shannon River	“*Westralunio carteri*” II	MZ668777 MZ668778 MZ668779 MZ668780 MZ668781	MZ668897 MZ668898 MZ668899 MZ668900 MZ668901	WAM S112755^28^ WAM S112756^28^ WAM S112757^28^ WAM S112758^28^ WAM S112759^28^
SHANNON, WA, Australia	Inlet River	“*Westralunio carteri*” II	MZ668817 MZ668818 MZ668819 MZ668820 MZ668821	MZ668937 MZ668938 MZ668939 MZ668940 MZ668941	WAM S112795^28^ WAM S112796^28^ WAM S112797^28^ WAM S112798^28^ WAM S112799^28^
SHANNON, WA, Australia	Deep River	“*Westralunio carteri*” II	MZ668782 MZ668783 MZ668784 MZ668785 MZ668786	MZ668902 MZ668903 MZ668904 MZ668905 MZ668906	WAM S112760^28^ WAM S112761^28^ WAM S112762^28^ WAM S112763^28^ WAM S112764^28^
SHANNON, WA, Australia	Walpole River	“*Westralunio carteri*” II	MZ668822 MZ668823 MZ668824 MZ668825 MZ668826	MZ668942 MZ668943 MZ668944 MZ668945 MZ668946	WAM S112800^28^ WAM S112801^28^ WAM S112802^28^ WAM S112803^28^ WAM S112804^28^
KENT COAST, WA, Australia	Bow River	“*Westralunio carteri*” II	MZ668787 MZ668788 MZ668789 MZ668790 MZ668791	MZ668907 MZ668908 MZ668909 MZ668910 MZ668911	WAM S112765^28^ WAM S112766^28^ WAM S112767^28^ WAM S112768^28^ WAM S112769^28^
KENT COAST, WA, Australia	Kent River	“*Westralunio carteri*” II	MT040636 MT040637 MT040638 MT040639 MT040640 MT040667 MT040668 MZ668792 MZ668793 MZ668794 MZ668795 MZ668796	MT040058 MT040059 – – – – – MZ668912 MZ668913 MZ668914 MZ668915 MZ668916	WAM S56205^27^ WAM S56206^27^ WAM S56207^27^ WAM S56208^27^ WAM S56209^27^ WAM S82758.1^27^ WAM S82758.2^27^ WAM S112770^28^ WAM S112771^28^ WAM S112772^28^ WAM S112773^28^ WAM S112774^28^
DENMARK COAST, WA, Australia	Marbellup Brook	“*Westralunio carteri*” II	MZ668837 MZ668838 MZ668839 MZ668840 MZ668841	MZ668957 MZ668958 MZ668959 MZ668960 MZ668961	WAM S112815^28^ WAM S112816^28^ WAM S112817^28^ WAM S112818^28^ WAM S112819^28^
ALBANY COAST, WA, Australia	Goodga River	“*Westralunio carteri*” II	MT040633 MT040634 MT040635 MT040669 MZ668722 MZ668723 MZ668724 MZ668725 MZ668726	– – – – MZ668842 MZ668843 MZ668844 MZ668845 MZ668846	WAM S56200^27^ WAM S56202^27^ WAM S56203^27^ WAM S82756^27^ WAM S112700^28^ WAM S112701^28^ WAM S112702^28^ WAM S112703^28^ WAM S112704^28^
ALBANY COAST, WA, Australia	Waychinicup River	“*Westralunio carteri*” II	MT040662 MT040663 MZ668827 MZ668828 MZ668829 MZ668830 MZ668831	MT040063 MT040064 MZ668947 MZ668948 MZ668949 MZ668950 MZ668951	WAM S66127^27^ WAM S66128^27^ WAM S112805^28^ WAM S112806^28^ WAM S112807^28^ WAM S112808^28^ WAM S112809^28^

Specimen provenance, GenBank accession numbers for mitochondrial Cytochrome c Oxidase Subunit I (COI) and 16S rDNA (16S) gene sequences, and voucher codes (in reference to source publications) are provided. Institution codes: *FMNH* Field Museum of Natural History, Chicago, Illinois, USA; *UMMZ* University of Michigan Museum of Zoology, Ann Arbor, Michigan, USA; *WAM* Western Australian Museum, Welshpool, Western Australia, Australia. *ESU* Evolutionary Significant Unit, *NSW* New South Wales, *WA* Western Australia.

#### Phylogenetic analyses and species delimitation

The COI alignment, including outgroups, was reduced to unique haplotypes using DnaSP v6^106^. Individual haplotype names correspond to those used in Benson et al.^28^. The alignment was partitioned by codon position in Mesquite version 3.61^107^, and the best fitting substitution model for each partition was identified by jModelTest^108^. This alignment was then used to construct Maximum likelihood (ML) and Bayesian inference (BI) trees in W-IQ-TREE^109^ and MrBayes version 3.2.7a^110^ respectively. The ML analysis was performed with 10,000 ultrafast bootstrap replicates using the standard settings of the selected substitution models. The BI analysis was performed with two independent runs of 1 × 10^7^ generations sampling every 500 generations to achieve an average standard deviation of the split frequencies that was consistently < 0.01, and ESS values > 200. Convergence of the MCMC chains was also confirmed in Tracer version 1.7.1^111^. In order to determine the number of distinct taxa within *W. carteri*, three methods of species delimitation were applied to the COI dataset, excluding outgroups. Firstly, the distance-based method, Assemble Species by Automatic Partitioning (ASAP)^112^, was implemented using the Jukes-Cantor (JC69) option on the online webserver (https://bioinfo.mnhn.fr/abi/public/asap/). Secondly, a statistical parsimony method was run in TCS 1.21^113^ using a 95% connection limit. Finally, the BI tree was assessed using Bayesian implementation of the Poisson Tree Process (bPTP)^114^ on the bPTP webserver (https://species.h-its.org/ptp/) with the maximum allowable number of MCMC generations (5 × 10^5^) and 20% burn-in. The ML and BI trees were edited in FigTree v1.4.4 (http://tree.bio.ed.ac.uk/software/figtree/) and Inkscape v1 (https://inkscape.org).

#### Molecular diagnosis

For the molecular diagnosis of taxa within *W. carteri* we considered fixed nucleotide differences found within the alignments of the full dataset for each gene (i.e., character attributes that were present in all individuals of one taxon while being absent in all individuals of the other two taxa^115–117^. For each gene, these characters were identified using the ‘toggle conserved sites’ option in MEGA X 10.1.8^105^. The uncorrected p-distance (mean and SE) to the nearest neighbour of each taxon was determined in the same software.

### Shell measurements

**Figure 8:**
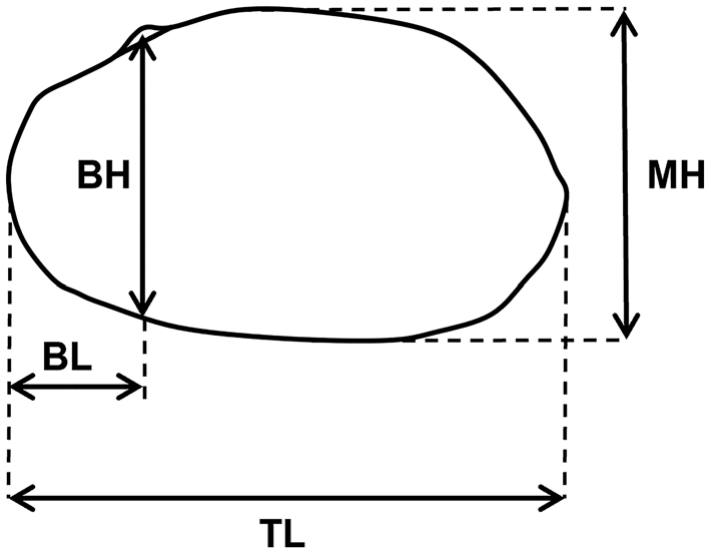
Measurement scheme used to quantify freshwater mussel size in this study, redrawn from McMichael & Hiscock^10^. *BH* beak height, *BL* beak length, *MH* maximum height, *TL* total length.

Shells were measured to the nearest 0.1 mm using manual callipers or, for photographed specimens, to the nearest 0.5 mm using a ruler as a scale bar appearing in each photograph. Shells utilised for Fourier shape analysis (see below) from photos provided by Graf & Cummings^63^ were not measured because scales were not included in the photos. For shell morphometry, we followed measurement procedures defined by McMichael & Hiscock^10^ (Fig. 8): total length (TL), measured as the horizontal distance from the anterior apex to the posterior apex of the valve; maximum height (MH), measured as the vertical distance from the ventral edge to the point of the beak; beak height (BH), measured from the point of the beak to the ventral margin; beak length (BL), measured as the horizontal distance from the anterior apex to the imaginary line perpendicular to the apex of the beak.

### Morphometric analysis

We conducted traditional morphometric analysis and outline (Fourier shape) analysis to assess whether shell shapes are different for each of the *Westralunio* ESUs identified^27,28^. Details of specimens used in these two analyses are provided in the ‘material examined’ sections below. Geographic distribution of these samples is illustrated in Fig. 6. A total of 446 and 411 specimens were included in traditional and Fourier shape analysis, respectively: 294 and 273 “*W. carteri*” I, respectively; 140 and 126 “*W. carteri*” II, respectively; and 12 and 12 “*W. carteri*” III, respectively.

Like Ponder & Bayer^11^ and Sheldon^101^, we tested for differences in shell measurements and sagittal shell shape between the two species and among the three taxa, respectively. ‘Traditional’ shell shape indices^10^ were calculated from shell measurements for maximum height index (MHI), beak height index (BHI), and beak length index (BLI), such that: $\mathrm{MHI}= \frac{\mathrm{MH}}{\mathrm{L}}$; $\mathrm{BHI}= \frac{\mathrm{BH}}{\mathrm{MH}}$; $\mathrm{BLI}= \frac{\mathrm{BL}}{\mathrm{L}}$. Firstly, significant differences in TL, MH, BH, BL, MHI, BHI and BLI, respectively, were tested for using ANOVAs followed by pairwise Tukey’s posthoc comparisons. Secondly, a Principal Component Analysis (PCA) on these variables was performed, following ANOVAs and Tukey’s posthoc comparisons on the first two PC-axes. Thirdly, we employed Discriminant Analysis (DA) to assess the proportion of specimens that would be assigned to the correct taxa based on these shell indices.

Overall shell shape was analysed using Fourier shape analysis^118^. This method breaks information on sagittal shell outlines of specimens into a set number of harmonics, each of which is explained by two Fourier coefficients, which are then analysed statistically. Specimens held in the Western Australian Museum collections were photographed using a Canon EOS 3D digital camera. A photographic stand was set up to hold the camera at the same distance, angle and focus for each photographed specimen. Black felt was used as a background medium to minimise shadows in the photos and a silver ruler was included in each photo in the same position as a scale bar. Additional digital photos of shell specimens from other museum collections were obtained, with permission, from the Mussel Project website^63^.

Shell outlines of specimens were digitised into xy-coordinates using the program IMAGEJ^119^. The digitised outlines were then subjected to Fast Fourier transformation using the program HANGLE, applying a smoothing normalisation of 20 to eliminate high frequency pixel noise. Preliminary analysis indicated that the first 10 harmonics described the outlines with sufficiently high precision. Discarding of the first harmonic, which did not contain any shape information, resulted in a set of 18 Fourier coefficients per individual. After rotating outlines to maximum overlap with program HTREE, a PCA was performed on the 18 Fourier coefficients using program PAST^120^. Synthetic outlines of extreme shell forms were drawn using program HCURVE^118^.

To test for statistical differences in overall sagittal shell shape between the two species (*W. carteri* and *W. inbisi*), we conducted t-tests on the first two PC-axes and carried out an analysis of similarities (ANOSIM; 9,999 permutations, Euclidean distance) and DA on the set of 18 Fourier coefficients. The degree of shell inflation (relative shell width) was not considered because, in contrast to sagittal shell shape, this morphological character is strongly influenced by ontogenetic growth in Unionida^14,73^. Statistical analyses were conducted in PAST 3.22^121^ (PCAs, DAs, ANOSIM), and R version 3.6.3 (ANOVAs and Tukey’s post hoc comparisons).

### Nomenclatural acts

The electronic edition of this article conforms to the requirements of the amended ICZN^44^.

## Supplementary Information

Supplementary Information 1.

Supplementary Table S1.

Supplementary Table S2.

Supplementary Table S3.

## Data Availability

Spatial data for mapping are provided in the Methods section. Data for specimen records we examined for this study (Tables 3, S1, S2 and S3) are available online. Those held at the Western Australian Museum Collection and Research Centre, Perth, WA, Australia (WAM) and the Australian Museum, Sydney, NSW, Australia are available from the Online Zoological Catalogue of Australian Museums (OZCAM) at https://ozcam.org.au/ and the Atlas of Living Australia (ALA) at https://www.ala.org.au/. Additional specimen records are available from the Mussel Project Website (Musselp) at https://mussel-project.uwsp.edu/index.html and individual museum collections as follows—University of Michigan Museum of Zoology, Ann Arbor, MI, USA (UMMZ): https://fms02.lsa.umich.edu/fmi/webd/ummz_mollusks; Field Museum of Natural History, Chicago, IL, USA (FMNH): https://www.fieldmuseum.org/science/research/area/invertebrates; The Natural History Museum, London, UK (BMNH): https://data.nhm.ac.uk/search#; Museum of Comparative Zoology, Harvard University, Cambridge, MA, USA (MCZ): https://mcz.harvard.edu/malacology-research-collection. The genetic sequences utilised for this study (Tables 3, S1, S2 and S3) are available from GenBank (https://www.ncbi.nlm.nih.gov/nuccore/?term=Westralunio+carteri). The dataset generated in this study is also available from the corresponding author on reasonable request.

## Abbreviations

AMS: The Australian Museum, Sydney, NSW, Australia
Bk: Brook
BMNH: The Natural History Museum, London, UK
FMNH: Field Museum of Natural History, Chicago, IL, USA
ICZN: International Code for Zoological Nomenclature
Lk: Lake
MCZ: Museum of Comparative Zoology, Harvard University, Cambridge, MA, USA
MU: Murdoch University, Murdoch, WA, Australia
R: River
Res: Reservoir
UMCZ: University Museum of Zoology Cambridge, UK
UMMZ: University of Michigan Museum of Zoology, Ann Arbor, MI, USA
WAM: Western Australian Museum, Perth, WA, Australia

## References

[1] Bickford D (2007). Cryptic species as a window on diversity and conservation. Trends. Ecol. Evol..

[2] Bolotov IN (2017). New taxa of freshwater mussels (Unionidae) from a species-rich but overlooked evolutionary hotspot in Southeast Asia. Sci. Rep..

[3] Bolotov IN (2019). Eight new freshwater mussels (Unionidae) from tropical Asia. Sci. Rep..

[4] Johnson NA (2018). Integrative taxonomy resolves taxonomic uncertainty for freshwater mussels being considered for protection under the U.S. endangered species act. Sci. Rep..

[5] Lopes-Lima M (2018). Expansion and systematics redefinition of the most threatened freshwater mussel family, the Margaritiferidae. Mol. Phylogenet. Evol..

[6] Lopes-Lima M (2020). Freshwater mussels (Bivalvia: Unionidae) from the rising sun (Far East Asia): phylogeny, systematics, and distribution. Mol. Phylogenet. Evol..

[7] Konopleva ES (2019). A new genus and two new species of freshwater mussels (Unionidae) from western Indochina. Sci. Rep..

[8] Smith CH, Johnson NA, Inoue K, Doyle RD, Randklev CR (2019). Integrative taxonomy reveals a new species of freshwater mussel, *Potamilus streckersoni* sp. nov. (Bivalvia: Unionidae): implications for conservation and management. Syst. Biodiv..

[9] Iredale T (1934). The freshwater mussels of Australia. Aust. Zool..

[10] McMichael DF, Hiscock ID (1958). A monograph of the freshwater mussels (Mollusca: Pelecypoda) of the Australian Region. Aust. J. Mar. Freshw. Res..

[11] Ponder WF, Bayer M (2004). A new species of *Lortiella* (Mollusca: Bivalvia: Unionoidea: Hyriidae) from northern Australia. Molluscan Res..

[12] Balla SA, Walker KF (1991). Shape variation in the Australian freshwater mussel *Alathyria jacksoni* Iredale (Bivalvia, Hyriidae). Hydrobiologia.

[13] Mock KF (2010). Genetic structuring in the freshwater mussel *Anodonta* corresponds with major hydrologic basins in the western United States. Mol. Ecol..

[14] Walker KF, Jones HA, Klunzinger MW (2014). Bivalves in a bottleneck: Taxonomy, phylogeography and conservation of freshwater mussels (Bivalvia: Unionoida) in Australasia. Hydrobiologia.

[15] Williams JD (2017). A revised list of the freshwater mussels (Mollusca: Bivalvia: Unionida) of the United States and Canada. Freshw. Mol. Biol. Conserv..

[16] Graf DL, Cummings KS (2021). A ‘big data’ approach to global freshwater mussel diversity (Bivalvia: Unionoida), with an updated checklist of genera and species. J. Mollus. Stud..

[17] Graf DL, Cummings KS (2006). Palaeoheterodont diversity (Mollusca: Trigonoida + Unionoida): What we know and what we wish we knew about freshwater mussel evolution. Zool. J. Linn. Soc. Lond..

[18] Graf DL, Cummings KS (2007). Review of the systematics and global diversity of freshwater mussel species (Bivalvia: Unionoida). J. Mollus. Stud..

[19] Bauer G, Wächtler K (2001). Ecology and Evolution of the Freshwater Mussels Unionoida.

[20] Marshall BA, Fenwick MC, Ritchie PA (2014). New Zealand recent Hyriidae (Mollusca: Bivalvia: Unionida). Molluscan Res..

[21] Graf DL, Jones HA, Geneva AJ, Pfeiffer JM, Klunzinger MW (2015). Molecular phylogenetic analysis supports a Gondwanan origin of the Hyriidae (Mollusca: Bivalvia: Unionida) and the paraphyly of Australasian taxa. Mol. Phylogenet. Evol..

[22] Santos-Neto GC (2016). Genetic relationships among freshwater mussel species from fifteen Amazonian rivers and inferences on the evolution of the Hyriidae (Mollusca: Bivalvia: Unionida). Mol. Phylogenet. Evol..

[23] Miyahira IC, Dos Santos SB, Mansur MCD (2017). Freshwater mussels from South America: State of the art of Unionida, specially Rhipidodontini. Biota Neotrop..

[24] Tapparone Canefri C (1883). Fauna malacologica della Nuova Guinea e delle isole adiaoenti. Ann. Mus. Civ. Stor. Nat. Genova.

[25] Clench W (1957). Two new land and freshwater mollusks from New Guinea. Breviora.

[26] Philippi, R. A. *Abbildungen und Beschreibungen neuer oder wenig gekaunter Conchylien.* Vol. 3, *Unio.* (Cassel, 1845–1851).

[27] Klunzinger MW (2021). Phylogeographic study of the West Australian freshwater mussel, *Westralunio carteri*, uncovers evolutionarily significant units that raise new conservation concerns. Hydrobiologia.

[28] Benson J, Stewart BA, Close PG, Lymbery AJ (2022). Evidence for multiple refugia and hotspots of genetic diversity for *Westralunio carteri*, a threatened freshwater mussel in south-western Australia. Aquat. Conserv..

[29] Klunzinger MW (2012). Distribution of *Westralunio carteri* Iredale, 1934 (Bivalvia: Unionoida: Hyriidae) on the south coast of south-western Australia, including new records of the species. J. R. Soc. West. Aust..

[30] Coates DJ, Byrne M, Moritz C (2018). Genetic diversity and conservation units: Dealing with the species-population continuum in the age of genomics. Front. Ecol. Evol..

[31] Linnaeus, C. *Systema Naturae per Regna Tria Naturae, Secundum Classes, Ordines, Genera, Species, cum Characteribus*, (Differentiis, Synonymis, locis 1: 823, emendanda, 1758).

[32] Grobben K (1894). Zur kenntnis der morphologie, der verwandtschaftsverhältnisse und des systems der mollusken. Sitzung. Kaiserl. Akad. Wissenschaften. Math. Nat. Classe..

[33] Gray JE (1854). A revision of the arrangement of the families of bivalve shells (Conchifera). Ann. Mag. Nat. Hist..

[34] Newell ND (1965). Classification of the Bivalvia. Am. Mus. Nov..

[35] Bouchet P, Rocroi JP (2010). Nomenclator of Bivalve Families; with a classification of bivalve families by Bieler, R., Carter, J. G. & Coan, E. V. Malacologia.

[36] Rafinesque CS (1820). Monographie des coquilles bivalves fluviatiles de la rivière Ohio, contenant douze genres et soixante-huit espèces. Ann. Gén. Sci. Phys..

[37] Parodiz JJ, Bonetto AA (1963). Taxonomy and zoogeographic relationships of the South American naiades (Pelecypoda: Unionacea and Mutelacea). Malacologia.

[38] Lamarck, J. B. P. *Histoire naturelle des animaux sans vertébres*. (Tome 6, lére partie, 1819).

[39] Menke CT (1843). Molluscorum Novae Hollandiae Specimen.

[40] Reeve, L. Monograph of the Genus *Unio.* In Reeve, L. & Sowerby, G.B. (eds.) *Conchologica Iconica* Vol. 16. (1864–1865).

[41] Smith, E. A. *The Zoology of the Voyage of H.M.S. Erebus and Terror.* Volume 2. *Mollusca.* (1874).

[42] Cotton BC, Gabriel CJ (1932). Australian Unionidae. Proc. R. Soc. Vic.

[43] Iredale T (1943). A basic list of the fresh-water Mollusca of Australia. Aust. Zool..

[44] ICZN (International Commission On Zoological Nomenclature). *International code of zoological nomenclature*. *Fourth edition adopted by the International Union of Biological Sciences* (International Trust for Zoological Nomenclature and The Natural History Museum, 1999).

[45] Ponder, W. F. *et al*. *Australian Freshwater Molluscs*. https://keys.lucidcentral.org/keys/v3/freshwater_molluscs/ (2020).

[46] Zieritz A, Sartori AF, Klunzinger MW (2013). Morphological evidence shows that not all Velesunioninae have smooth umbos. J. Mollus. Stud..

[47] Klunzinger MW, Beatty SJ, Morgan DL, Thomson GJ, Lymbery AJ (2012). Glochidia ecology in wild fish populations and laboratory determination of competent host fishes for an endemic freshwater mussel of south-western Australia. Aust. J. Zool..

[48] Klunzinger MW, Thomson GJ, Beatty SJ, Morgan DL, Lymbery AJ (2013). Morphological and morphometrical description of the glochidia of *Westralunio carteri* Iredale, 1934 (Bivalvia: Unionoida: Hyriidae). Molluscan Res..

[49] Walker KF (2017). Reproductive phenology of river and lake populations of freshwater mussels (Unionida: Hyriidae) in the River Murray. Molluscan Res..

[50] Klunzinger, M. W. *Ecology, Life History and Conservation Status of* Westralunio carteri *Iredale, 1934, an Endemic Freshwater Mussel of South-western Australia*. PhD Thesis, Murdoch University (2012).

[51] Sauvage HE (1880). Description des gobioides nouveaux ou peu connus de la collection du Muséum d'Histoire Naturelle. B. Soc. Phylom. Par..

[52] Girard CF (1859). Ichthyological notices. Proc. Acad. Nat. Sci. Phila..

[53] Castelnau FL (1873). Contribution to the ichthyology of Australia. 8. Fishes of Western Australia. Proc. Zool. Acclim. Soc. Vic..

[54] Whitley GP (1944). New sharks and fishes from Western Australia. Aust. Zool..

[55] Quoy, J. R. C. & Gaimard, J. P. Description des Poissons. Chapter IX. In Freycinet, L. de, Voyage autour du Monde...exécuté sur les corvettes de L. M. "L'Uranie" et "La Physicienne," pendant les années 1817, 1818, 1819 et 1820. Paris. 192–401 [1–328 in 1824; 329–616 in 1825], Atlas pls. 43–65 (1824–1825).

[56] Ogilby JD (1899). Contribution to Australian ichthyology. Proc. Linn. Soc. NSW.

[57] Prince JD, Ivantsoff W, Potter LC (1982). *Atherinosoma wallacei*, a new species of estuarine and inland water silverside (Teleostei: Atherinidae) from the Swan-Avon and Murray Rivers, Western Australia. Aust. Zool..

[58] Hensel R (1868). Beiträge zur kenntniss der wirbelthiere südbrasiliens (fortsetzung). Arch. Nat..

[59] Klunzinger MW, Beatty SJ, Morgan DL, Lymbery AJ, Haag WR (2014). Age and growth in the Australian freshwater mussel, *Westralunio carteri*, with an evaluation of the fluorochrome calcein for validating the assumption of annulus formation. Freshw. Sci..

[60] Guerra D (2019). Variability of mitochondrial ORFans hints at possible differences in the system of doubly uniparental inheritance of mitochondria among families of freshwater mussels. BMC Ecol. Biol..

[61] Klunzinger MW, Beatty SJ, Morgan DL, Pinder AM, Lymbery AJ (2015). Range decline and conservation status of *Westralunio carteri* Iredale, 1934 (Bivalvia: Hyriidae) from south-western Australia. Aust. J. Zool..

[62] Küster, H. C. Die Flussperlmuscheln (*Unio* et *Hyria*). *Systematisches Conchylien-Cabinet von Martini und Chemnitz Fortgesetzt von Hofrath Dr. G. v. Schubert und Professor Dr. J.A. Wagner* **9,** 161–256, pl. 35, 46–86 (1861).

[63] Graf, D. L. & Cummings, K. S. *The Freshwater Mussels (Unionoida) of the World (and Other Less Consequential Bivalves). MUSSEL Project Web Site*. http://www.mussel-project.net/ (2018).

[64] Simpson, C. T. *A Descriptive Catalogue of the Naiades, or Pearly Fresh-Water Mussels*. Parts I–III. (1914).

[65] Haas F (1924). Unsere bisherigen Kenntnisse der Najadenfauna Neu-Guineas. Nova Guinea.

[66] McMichael DF (1956). Notes on the fresh water mussels of New Guinea. Nautilus.

[67] Bindon P, Chadwick R (2011). A Nyoongar Wordlist: From the South-west of Western Australia.

[68] Benson JA, Close PG, Stewart BA, Lymbery AJ (2018). Upstream recolonization by freshwater mussels (Unionoida: Hyriidae) following installation of a fishway. Aquat. Conserv..

[69] Benson JA, Close PG, Stewart BA, Lymbery AJ (2019). Freshwater tributaries provide refuge and recolonization opportunities for mussels following salinity reversal. Sci. Total Environ..

[70] Carstens BC, Pelletier TA, Reid NM, Satler JD (2013). How to fail at species delimitation. Mol. Ecol..

[71] Morrison CL (2021). Genetic and morphological characterization of the freshwater mussel clubshell species complex (*Pleurobema clava* and *Pleurobema oviforme*) to inform conservation planning. Ecol. Evol..

[72] Mansur MCD, Pimpão DM (2008). *Triplodon chodo*, a new species of pearly fresh water mussel from the Amazon Basin (Mollusca: Bivalvia: Unionoida: Hyriidae). Rev. Bras. Zool..

[73] Zieritz A, Aldridge DC (2009). Identification of ecophenotypic trends within three European freshwater mussel species (Bivalvia: Unionoida) using traditional and modern morphometric techniques. Biol. J. Linn. Soc Lond..

[74] Zieritz A, Hoffman JI, Amos W, Aldridge DC (2010). Phenotypic plasticity and genetic isolation-by-distance in the freshwater mussel *Unio pictorum* (Mollusca: Unionoida). Evol. Ecol..

[75] Garnett ST, Christidis L (2017). Taxonomy anarchy hampers conservation. Nature.

[76] Wu R, Liu X, Ouyang S, Wu X (2020). Comparative analyses of the complete mitochondrial genomes of three *Lamprotula* (bivalvia: unionidae) species: insight into the shortcomings of mitochondrial DNA for recently diverged species delimitation. Malacologia.

[77] Zachos FE (2016). Species Concepts in Biology: Historical Development, Theoretical Foundations, and Practical Relevance.

[78] Zachos FE (2018). (New) Species concepts, species delimitation and the inherent limitations of taxonomy. J. Genet..

[79] Mayr E, Ashlock PD (1991). Principles of Systematic Biology.

[80] Páll-Gergely B, Asami T, Sólymos P (2019). Subspecies description rates are higher in morphologically complex land snails. Zool. Scr..

[81] Simpson CT (1900). Synopsis of the naiades, or pearly fresh-water mussels. Proc. US Nat. Mus..

[82] Ortmann AE (1921). South American naiades, a contribution to the knowledge of the fresh-water mussels of South America. Mem. Carn. Mus..

[83] Morretes FL (1949). Ensaio do catálogo dos moluscos do Brasil. Arq. Mus. Paran..

[84] Parodiz JJ (1968). Annotated catalogue of the genus *Diplodon* (Unionacea-Hyriidae). Sterkiana.

[85] Haas F, Martens R, Hennig W, Wermuth H (1969). Superfamilia Unionacea. Das Tierrich Lieferung.

[86] Fenwick MC, Marshall BA (2006). A new species of *Echyridella* from New Zealand, and recognition of *Echyridella lucasi* (Suter, 1905) (Mollusca: Bivalvia: Hyriidae). Molluscan Res..

[87] Simone, L. R. L. *Land and Freshwater Molluscs of Brazil* (EGB, Fapesp, São Paulo, 2006).

[88] Lydeard C (2004). The global decline of nonmarine mollusks. Bioscience.

[89] Lopes-Lima M (2018). Conservation of freshwater bivalves at the global scale: Diversity, threats and research needs. Hydrobiologia.

[90] Ferreira-Rodríguez N (2019). Research priorities for freshwater mussel conservation assessment. Biol. Conserv..

[91] Benson J, Stewart BA, Close PG, Lymbery AJ (2021). Freshwater mussels in Mediterranean-climate regions: Species richness, conservation status, threats and conservation actions needed. Aquat. Conserv..

[92] Hughes J (2004). Past and present patterns of connectivity among populations of four cryptic species of freshwater mussels *Velesunio* spp. (Hyriidae) in central Australia. Mol. Ecol..

[93] Jones HA, Simpson RD, Humphrey CL (1986). The reproductive cycles and glochidia of freshwater mussels (Bivalvia: Hyriidae) of the Macleay River, northern New South Wales, Australia. Malacologia.

[94] Gray JE (1834). Proceedings for July 8th, 1834. Proc. Zool. Soc. Lond..

[95] Pimpão DM, Dreher Mansur MC, Aydos Bergonci PE, Beasley CR (2012). Comparative morphometry and morphology of glochidial shells of Amazonian Hyriidae (Mollusca: Bivalvia: Unionida). Am. Malac. Bull..

[96] Melchior M, Collier KJ, Clearwater SJ (2021). First record of complex release strategies and morphometry of glochidia in sympatric *Echyridella* species (Bivalvia: Unionida: Hyriidae). Hydrobiologia.

[97] Gray JE (1843). Travels in New Zealand.

[98] Morgan DL (2014). An overview of the ‘freshwater fishes’ of Western Australia. J. R. Soc. West. Aust..

[99] Buckley SJ (2018). Phylogenomic history of enigmatic pygmy perches: implications for biogeography, taxonomy and conservation. R. Soc. Open Sci..

[100] Baker AM, Sheldon F, Somerville J, Walker KF, Hughes JM (2004). Mitochondrial DNA phylogenetic structuring suggests similarity between two morphometrically plastic genera of Australian freshwater mussels (Unionoida: Hyriidae). Mol. Phylogenet. Evol..

[101] Sheldon F (2017). Variable plasticity in shell morphology of some Australian freshwater mussels (Unionoida, Hyriidae). Trans. R. Soc. S. Aust..

[102] AWRC. *Review of Australia’s Water Resources* 1975. (Australian Water Resources Council (AWRC), Department of Natural Resources, 1976).

[103] Geoscience Australia. *GEODATA TOPO 250K, Series 2*. (National Mapping Division, Commonwealth of Australia Department of Industry, Tourism and Resources, 2003).

[104] Hall TA (1999). BioEdit: A user-friendly biological sequence alignment editor and analysis program for Windows 95/98/NT. Nucleic Acids Symp. Ser..

[105] Kumar S, Stecher G, Li M, Knyaz C, Tamura K (2018). MEGA X: Molecular evolutionary genetics analysis across computing platforms. Mol. Biol. Evol..

[106] Rozas J (2017). DnaSP 6: DNA sequence polymorphism analysis of large data sets. Mol. Biol. Evol..

[107] Maddison, W. P. & Maddison, D. R. *Mesquite: A Modular System for Evolutionary Analysis. Version 3.61*. http://www.mequiteproject.org (2019).

[108] Darriba D, Taboada GL, Doallo R, Posada D (2012). jModelTest 2: More models, new heuristics and parallel computing. Nat. Method.

[109] Trifinopoulos J, Nguyen L, von Haeseler A, Minh BQ (2016). W-IQ-TREE: A fast online phylogenetic tool for maximum likelihood analysis. Nucleic Acids Res..

[110] Ronquist F (2012). MrBayes 3.2: Efficient Bayesian phylogenetic inference and model choice across a large model space. Syst. Biol..

[111] Rambaut A, Drummond AJ, Xie D, Baele G, Suchard MA (2018). Posterior summarisation in Bayesian phylogenetics using Tracer 1.7. Syst. Biol..

[112] Puillandre N, Brouillet S, Achaz G (2021). ASAP: Assemble species by automatic partitioning. Mol. Ecol. Res..

[113] Clement M, Posada D, Crandall KA (2000). TCS: A computer program to estimate gene genealogies. Mol. Ecol..

[114] Zhang J, Kapli P, Pavlidis P, Stamatakis A (2013). A general species delimitation method with applications to phylogenetic placements. Bioinformatics.

[115] Jörger KM, Schrödl M (2013). How to describe a cryptic species? Practical challenges of molecular taxonomy. Front. Zool..

[116] Renner SS (2016). A return to Linnaeus’s focus on diagnosis, not description: The use of DNA characters in the formal naming of species. Syst. Biol..

[117] Delić T, Trontelj P, Rendoš M, Fišer C (2017). The importance of naming cryptic species and the conservation of endemic subterranean amphipods. Sci. Rep..

[118] Crampton JS, Haines AJ (1996). Users’ manual for programs HANGLE, HMATCH and HCURVE for the Fourier shape analysis of two-dimensional outlines. Inst. Geol. Nucl. Sci. Sci. Rep..

[119] Rasband, W. ImageJ. *Image Processing and Analysis in Java*. http://rsbweb.nih.gov/ij (2008).

[120] Hammer, Ø. & Harper, D. A. T. *PAST Version 1.57*. http://folk.uio.no/ohammer/past/ (2006).

[121] Hammer Ø, Harper DAT, Ryan PD (2001). PAST: Paleontological statistics software package for education and data analysis. Palaeontol. Elec..

[122] Bolotov IN (2020). New freshwater mussel taxa discoveries clarify biogeographic division of Southeast Asia. Sci. Rep..

