# Author Correction: Integrated taxonomy reveals new threatened freshwater mussels (Bivalvia: Hyriidae: *Westralunio*) from southwestern Australia

**DOI:** 10.1038/s41598-023-40483-0

**Published:** 2023-08-22

**Authors:** Michael W. Klunzinger, Corey Whisson, Alexandra Zieritz, Justin A. Benson, Barbara A. Stewart, Lisa Kirkendale

**Affiliations:** 1 Australian Rivers Institute, Griffith University, Nathan, QLD 6111 Australia; 2 Mollusc Section, Department of Aquatic Zoology, Western Australian Museum, Welshpool, WA 6163 Australia; 3 School of Geography, University of Nottingham, University Park, Nottingham, NG7 2RD UK; 4 Centre for Natural Resource Management, UWA School of Agriculture and the Environment, The University of Western Australia, Albany, WA 6330 Australia

Correction to: *Scientific Reports* 10.1038/s41598-022-24767-5, published online 27 November 2022

The original version of this Article contained an error in Figure 4C, where the incorrect locality details for the type locality for *Westralunio inbisi meridiemus* were provided. As a result, the legend of Figure 4C,

“(**C**) Margaret River, Western Australia, type locality for *W. inbisi meridiemus*, at Canebreak Pool. Photo by Dr Michael W. Klunzinger.”

now reads:

“(**C**) Margaret River, Western Australia, type locality for *W. inbisi meridiemus*, at Apex Weir. Photo by Dr Michael W. Klunzinger.”

The original Figure 4 and accompanying legend appear below.

**Figure 4:**
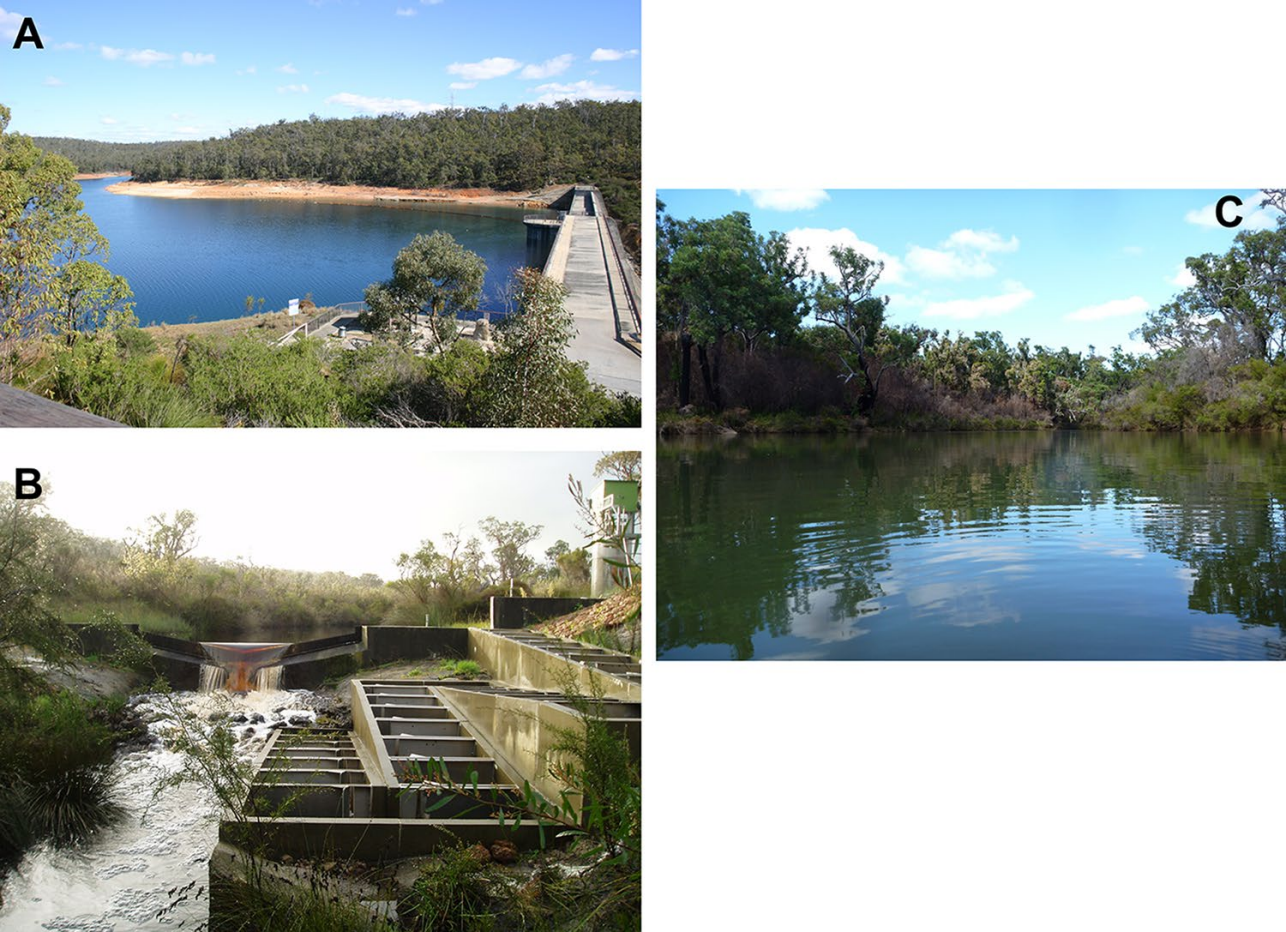
(**A**) Victoria Reservoir, Canning River, near Perth, Western Australia, type locality for *W. carteri*. Photo by Corey Whisson. (**B**) Goodga River, Western Australia, type locality for *W. inbisi inbisi*, at vertical slot fishway where holotype of *W.inbisi inbisi* was collected from. Photo provided with permission by Dr Stephen J. Beatty. (**C**) Margaret River, Western Australia, type locality for *W. inbisi meridiemus*, at Canebreak Pool. Photo by Dr Michael W. Klunzinger.

Furthermore, in the Results, under the subheading ‘Taxonomic accounts’, for the type locality of *Westralunio inbisi meridiemus* subsp. nov.,

“Canebreak Pool, Margaret River, Western Australia (33.9460°S, 115.0707°E, GDA94) (see Fig. 4C).”

now reads:

“Apex Weir, Margaret River, Western Australia (33.942995°S, 115.073151°E, GDA94) (see Fig. 4C).”

Additionally, Table 3 contained errors, where four voucher specimen numbers for “*Westralunio carteri*” II and III for “BLACKWOOD, WA, Australia, Chapman River”, located in column ‘Voucher/source’, were incorrect. Lastly, GenBank accession number “MT040660” was placed incorrectly on the same line as “MT040659” for “*Westralunio carteri*” II under “BLACKWOOD, WA, Australia, St. Johns Brook”. The correct and incorrect values appear below.

Incorrect:

**Table:**
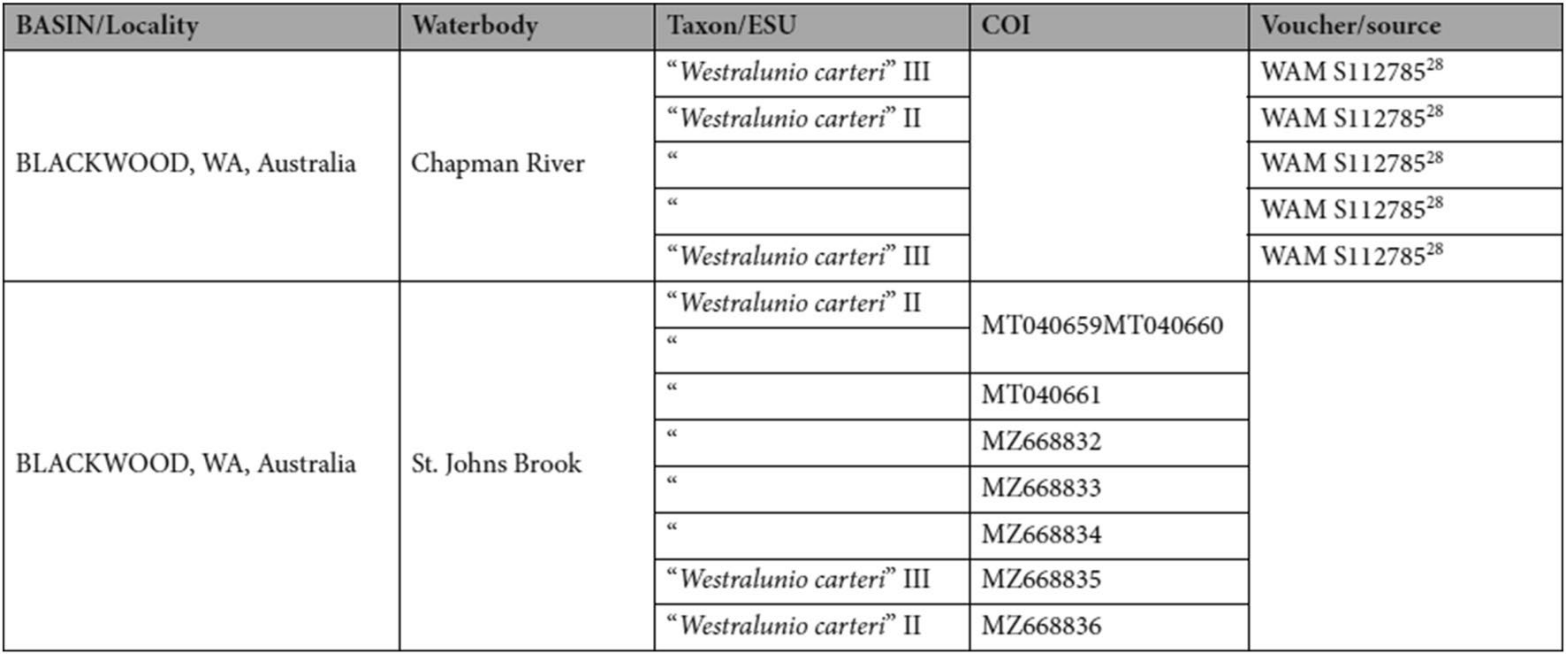

Correct:

**Table:**
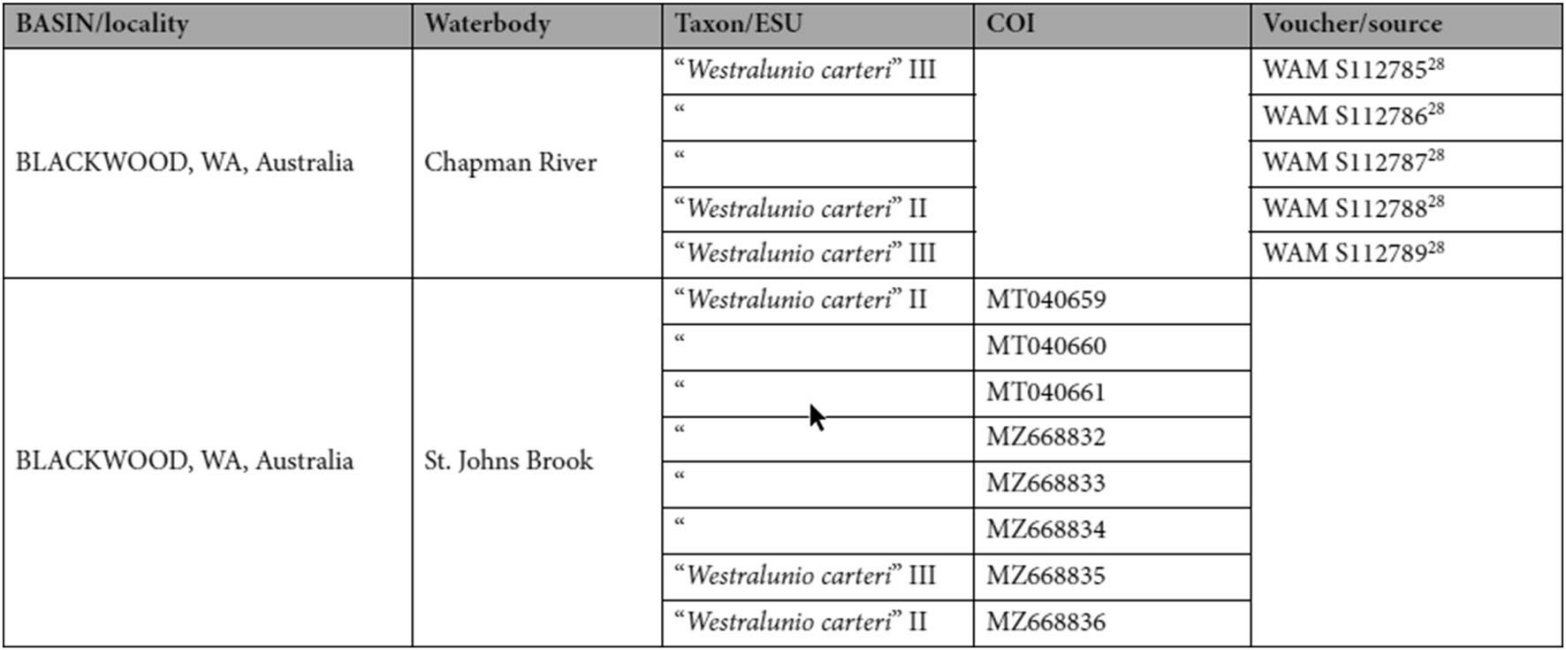

The original Article has been corrected.

