# Supplementary material for: Integrated taxonomy reveals new threatened freshwater mussels (Bivalvia: Hyriidae: Westralunio) from southwestern Australia
Source: Sci Rep. 2022 Nov 27;12:20385. doi: 10.1038/s41598-022-24767-5 (PMC9701689; doi:10.1038/s41598-022-24767-5)
Supplement: Supplementary file 1 — Supplementary Information 1. [file 41598_2022_24767_MOESM1_ESM.pdf]

Species delimitation outputs:

1. ASAP results:

Partition 1

Score: 1

Proba: 5.588822e-01

nb groups:3 (2)

Group[1] n: 14 ;id: H1 H6 H2 H3 H10 H7 H8 H14 H9 H11 H13 H5 H4 H12

Group[2] n: 8 ;id: H16 H15 H17 H18 H19 H20 H22 H21

Group[3] n: 24 ;id: H23 H27 H37 H30 H34 H33 H32 H40 H39 H43 H42 H41 H24 H29 H25 H26 H28 H31 H38 H45 H35 H36 H44 H46

See ASAP group-score tree.svg for the most likely number of groups (scores ranked from 1-5).

2. bPTP results:

Accptance rate: 0.86492599999999997

Merge: 249759

Split: 250241

Estimated number of species is between 8 and 43

Mean: 28.03

Max likilhood partition

Species 1 (support = 0.346)

H35,H36,H44

Species 2 (support = 0.995)

H42

Species 3 (support = 0.951)

H30

Species 4 (support = 0.982)

H1

Species 5 (support = 0.350)

H2,H3,H10

Species 6 (support = 0.914)

H31

Species 7 (support = 0.895)

H16

Species 8 (support = 0.776)

H15

Species 9 (support = 0.667)

H17

Species 10 (support = 0.890)

H23

Species 11 (support = 0.564)

H18

Species 12 (support = 0.480)

H5,H4

Species 13 (support = 0.231)

H20,H22

Species 14 (support = 0.803)

H7

Species 15 (support = 0.690)

H8

Species 16 (support = 0.586)

H6

Species 17 (support = 0.121)

H11,H12,H13,H14

Species 18 (support = 0.475)

H9

Species 19 (support = 0.275)

H24,H29

Species 20 (support = 0.527)

H25

Species 21 (support = 0.126)

H34,H37,H38,H39,H40,H43,H41,H45

Species 22 (support = 0.315)

H32,H46

Species 23 (support = 0.604)

H33

Species 24 (support = 0.253)

H19

Species 25 (support = 0.253)

H21

Species 26 (support = 0.265)

H28,H27

Species 27 (support = 0.527)

H26

3. TCS results:

OUTGROUP WEIGHTS FOR THREE GROUPS:

*** Network 1

H5 weigth = 0.07017543859649122

H4 weigth = 0.017543859649122806

H12 weigth = 0.017543859649122806

H11 weigth = 0.017543859649122806

H7 weigth = 0.10526315789473684

H2 weigth = 0.07017543859649122

H3 weigth = 0.14035087719298245

H1 weigth = 0.017543859649122806

H6 weigth = 0.2807017543859649

H10 weigth = 0.017543859649122806

H8 weigth = 0.10526315789473684

H14 weigth = 0.10526315789473684

H9 weigth = 0.017543859649122806

H13 weigth = 0.017543859649122806

Total weight = 28.5

Biggest outgroup probability is H6 (0.2807017543859649)

*** Network 2

H16 weigth = 0.047619047619047616

H15 weigth = 0.38095238095238093

H17 weigth = 0.047619047619047616

H18 weigth = 0.047619047619047616

H22 weigth = 0.047619047619047616

H21 weigth = 0.047619047619047616

H19 weigth = 0.19047619047619047

H20 weigth = 0.19047619047619047

Total weight = 10.5

Biggest outgroup probability is H15 (0.38095238095238093)

*** Network 3

H35	weigth = 0.08955223880597014

H36	weigth = 0.014925373134328358

H44	weigth = 0.014925373134328358

H45	weigth = 0.014925373134328358

H46	weigth = 0.014925373134328358

H41	weigth = 0.014925373134328358

H43	weigth = 0.014925373134328358

H38	weigth = 0.014925373134328358

H33	weigth = 0.014925373134328358

H32	weigth = 0.08955223880597014

H31	weigth = 0.014925373134328358

H30	weigth = 0.014925373134328358

H26	weigth = 0.014925373134328358

H24	weigth = 0.05970149253731343

H29	weigth = 0.014925373134328358

H34	weigth = 0.14925373134328357

H28	weigth = 0.014925373134328358

H25	weigth = 0.014925373134328358

H23	weigth = 0.014925373134328358

H27	weigth = 0.08955223880597014

H37	weigth = 0.208955223880597

H40	weigth = 0.014925373134328358

H39	weigth = 0.014925373134328358

H42	weigth = 0.05970149253731343

Total weight = 33.5

Biggest outgroup probability is H37	(0.208955223880597)

PARSIMONY PROBABILITY

For 1 step(s),	P(95%) = 0.9993189000594782

For 2 step(s),	P(95%) = 0.9974565799863522

For 3 step(s),   P(95%) = 0.9947317351868009

For 4 step(s),   P(95%) = 0.9911062827467767

For 5 step(s),   P(95%) = 0.9865849716570294

For 6 step(s),   P(95%) = 0.9811775162679158

For 7 step(s),   P(95%) = 0.9748957304418876

For 8 step(s),   P(95%) = 0.9677537854640341

For 9 step(s),   P(95%) = 0.9597681955032399

For 10 step(s),   P(95%) = 0.9509577530083807

For 11 step(s),   P(95%) = 0.9413434557124126

RUN SETTINGS

Calculated maximum connection steps at 95% = 10

Gaps treated as fifth state

HAPLOTYPES

Number of haplotypes = 46

Haplotype list:

- H1 :
- H2 :
- H3 :
- H5 :
- H4 :
- H7 :
- H8 :
- H6 :
- H10 :
- H9 :
- H11 :
- H12 :
- H13 :
- H14 :
- H16 :

- H15 :
- H17 :
- H19 :
- H18 :
- H23 :
- H24 :
- H25 :
- H26 :
- H20 :
- H28 :
- H27 :
- H21 :
- H29 :
- H22 :
- H30 :
- H31 :
- H33 :
- H32 :
- H34 :
- H35 :
- H36 :
- H37 :
- H38 :
- H39 :
- H40 :
- H43 :
- H42 :
- H41 :
- H44 :
- H46 :
- H45 :

CONNECTIONS

adding two min taxa 3[H5] , 1[H2]

In clusters 3 , 11 distance 2

adding two min taxa 3[H5] , 7[H6]

In clusters 3 , 11 distance 2

adding two min taxa 15[H15] , 23[H20]

In clusters 14 , 28 distance 2

adding two min taxa 34[H35] , 41[H42]

In clusters 34 , 45 distance 2

REAL DISTANCE MATRIX

pos: 1 2 3 4 5 6 7 8 9 10 11 12 13 14 15 16 17 18 19 20 21 22 23 24 25 26 27 28 29 30 31 32 33 34 35 36 37 38 39 40 41 42 43 44 45 46

[H1]1 : -- 3 2 3 4 2 3 1 3 2 2 3 2 2 ##

[H2]2 : 3 -- 1 2 3 3 4 2 2 3 3 4 3 3 ##

[H3]3 : 2 1 -- 3 4 2 3 1 1 2 2 3 2 2 ##

[H5]4 : 3 2 3 -- 1 3 4 2 4 3 3 4 3 3 ##

[H4]5 : 4 3 4 1 -- 3 4 3 5 4 4 5 4 4 ##

[H7]6 : 2 3 2 3 3 -- 1 1 3 2 2 3 2 2 ##

[H8]7 : 3 4 3 4 4 1 -- 2 4 3 3 4 3 1 ##

[H6]8 : 1 2 1 2 3 1 2 -- 2 1 1 2 1 1 ##

[H10]9 : 3 2 1 4 5 3 4 2 -- 3 3 4 3 3 ##

[H9]10 : 2 3 2 3 4 2 3 1 3 -- 2 3 2 2 ##

[H11]11 : 2 3 2 3 4 2 3 1 3 2 -- 3 2 2 ##

[H12]12 : 3 4 3 4 5 3 4 2 4 3 3 -- 3 3 ##

[H13]13 : 2 3 2 3 4 2 3 1 3 2 2 3 -- 2 ##

[H14]14 : 2 3 2 3 4 2 1 1 3 2 2 3 2 -- ##

[H16]15 : ## ## ## ## ## ## ## ## ## ## ## ## ## ## -- 1 2 4 2 ## ## ## ## 3 ## ## 8 ## 5 ## ## ## ## ## ## ## ## ## ## ## ## ## ## ## ## ##

[H15]16 : ## ## ## ## ## ## ## ## ## ## ## ## ## ## 1 -- 1 3 1 ## ## ## ## 2 ## ## 7 ## 4 ## ## ## ## ## ## ## ## ## ## ## ## ## ## ## ## ##

[H17]17 : ## ## ## ## ## ## ## ## ## ## ## ## ## ## 2 1 -- 4 2 ## ## ## ## 3 ## ## 8 ## 5 ## ## ## ## ## ## ## ## ## ## ## ## ## ## ## ## ##

[H19]18 : ## ## ## ## ## ## ## ## ## ## ## ## ## ## 4 3 4 -- 4 ## ## ## ## 1 ## ## 4 ## 3 ## ## ## ## ## ## ## ## ## ## ## ## ## ## ## ## ##

[H18]19 : ## ## ## ## ## ## ## ## ## ## ## ## ## ## 2 1 2 4 -- ## ## ## ## 3 ## ## 8 ## 5 ## ## ## ## ## ## ## ## ## ## ## ## ## ## ## ## ##

[H23]20 : ## ## ## ## ## ## ## ## ## ## ## ## ## ## ## ## ## ## ## -- 3 3 3 ## 3 1 ## 4 ## 4 5 5 4 3 5 6 2 5 3 4 3 3 3 6 7 5

[H24]21 : ## ## ## ## ## ## ## ## ## ## ## ## ## ## ## ## ## ## ## 3 -- 2 2 ## 4 2 ## 1 ## 3 4 4 3 2 6 7 3 4 4 3 4 4 4 7 6 4

[H25]22 : ## ## ## ## ## ## ## ## ## ## ## ## ## ## ## ## ## ## ## 3 2 -- 4 ## 4 2 ## 3 ## 5 6 6 5 4 6 7 3 6 4 5 4 4 4 7 8 6

[H26]23 : ## ## ## ## ## ## ## ## ## ## ## ## ## ## ## ## ## ## ## 3 2 4 -- ## 4 2 ## 3 ## 3 4 4 3 2 6 7 3 4 4 3 4 4 4 7 6 4

[H20]24 : ## ## ## ## ## ## ## ## ## ## ## ## ## ## 3 2 3 1 3 ## ## ## ## -- ## ## 5 ## 2 ## ## ## ## ## ## ## ## ## ## ## ## ## ## ## ## ##

[H28]25 : ## ## ## ## ## ## ## ## ## ## ## ## ## ## ## ## ## ## ## 3 4 4 4 ## -- 2 ## 5 ## 5 6 6 5 4 6 7 3 6 4 5 4 4 4 7 8 6

[H27]26 : ## ## ## ## ## ## ## ## ## ## ## ## ## ## ## ## ## ## ## 1 2 2 2 ## 2 -- ## 3 ## 3 4 4 3 2 4 5 1 4 2 3 2 2 2 5 6 4

[H21]27 : ## ## ## ## ## ## ## ## ## ## ## ## ## ## 8 7 8 4 8 ## ## ## ## 5 ## ## -- ## 7 ## ## ## ## ## ## ## ## ## ## ## ## ## ## ## ## ##

[H29]28 : ## ## ## ## ## ## ## ## ## ## ## ## ## ## ## ## ## ## ## 4 1 3 3 ## 5 3 ## -- ## 4 5 5 4 3 7 8 4 5 5 4 5 5 5 8 7 5

[H22]29 : ## ## ## ## ## ## ## ## ## ## ## ## ## ## 5 4 5 3 5 ## ## ## ## 2 ## ## 7 ## -- ## ## ## ## ## ## ## ## ## ## ## ## ## ## ## ## ##

[H30]30 : ## ## ## ## ## ## ## ## ## ## ## ## ## ## ## ## ## ## ## 4 3 5 3 ## 5 3 ## 4 ## -- 3 3 2 1 5 6 2 3 3 2 3 3 3 6 5 3

[H31]31 : ## ## ## ## ## ## ## ## ## ## ## ## ## ## ## ## ## ## ## 5 4 6 4 ## 6 4 ## 5 ## 3 -- 4 3 2 6 7 3 4 4 3 4 4 4 7 4 4

[H33]32 : ## ## ## ## ## ## ## ## ## ## ## ## ## ## ## ## ## ## ## 5 4 6 4 ## 6 4 ## 5 ## 3 4 -- 1 2 6 7 3 4 4 3 4 4 4 7 4 4

[H32]33 : ## ## ## ## ## ## ## ## ## ## ## ## ## ## ## ## ## ## ## 4 3 5 3 ## 5 3 ## 4 ## 2 3 1 -- 1 5 6 2 3 3 2 3 3 3 6 3 3

[H34]34 : ## ## ## ## ## ## ## ## ## ## ## ## ## ## ## ## ## ## ## 3 2 4 2 ## 4 2 ## 3 ## 1 2 2 1 -- 4 5 1 2 2 1 2 2 2 5 4 2

[H35]35 : ## ## ## ## ## ## ## ## ## ## ## ## ## ## ## ## ## ## ## 5 6 6 6 ## 6 4 ## 7 ## 5 6 6 5 4 -- 1 3 4 4 5 4 2 4 1 8 6

[H36]36 : ## ## ## ## ## ## ## ## ## ## ## ## ## ## ## ## ## ## ## 6 7 7 7 ## 7 5 ## 8 ## 6 7 7 6 5 1 -- 4 5 5 6 5 3 4 2 9 7

[H37]37 : ## ## ## ## ## ## ## ## ## ## ## ## ## ## ## ## ## ## ## 2 3 3 3 ## 3 1 ## 4 ## 2 3 3 2 1 3 4 -- 3 1 2 1 1 1 4 5 3

[H38]38 : ## ## ## ## ## ## ## ## ## ## ## ## ## ## ## ## ## ## ## 5 4 6 4 ## 6 4 ## 5 ## 3 4 4 3 2 4 5 3 -- 4 3 4 2 4 5 6 4

[H39]39 : ## ## ## ## ## ## ## ## ## ## ## ## ## ## ## ## ## ## ## 3 4 4 4 ## 4 2 ## 5 ## 3 4 4 3 2 4 5 1 4 -- 3 2 2 2 5 6 4

[H40]40 : ## ## ## ## ## ## ## ## ## ## ## ## ## ## ## ## ## ## ## 4 3 5 3 ## 5 3 ## 4 ## 2 3 3 2 1 5 6 2 3 3 -- 3 3 3 6 5 3

[H43]41 : ## ## ## ## ## ## ## ## ## ## ## ## ## ## ## ## ## ## ## 3 4 4 4 ## 4 2 ## 5 ## 3 4 4 3 2 4 5 1 4 2 3 -- 2 2 5 6 4

[H42]42 : ## ## ## ## ## ## ## ## ## ## ## ## ## ## ## ## ## ## ## 3 4 4 4 ## 4 2 ## 5 ## 3 4 4 3 2 2 3 1 2 2 3 2 -- 2 3 6 4

[H41]43 : ## ## ## ## ## ## ## ## ## ## ## ## ## ## ## ## ## ## ## 3 4 4 4 ## 4 2 ## 5 ## 3 4 4 3 2 4 4 1 4 2 3 2 2 -- 5 6 4

[H44]44 : ## ## ## ## ## ## ## ## ## ## ## ## ## ## ## ## ## ## ## 6 7 7 7 ## 7 5 ## 8 ## 6 7 7 6 5 1 2 4 5 5 6 5 3 5 -- 9 7

[H46]45 : ## ## ## ## ## ## ## ## ## ## ## ## ## ## ## ## ## ## ## 7 6 8 6 ## 8 6 ## 7 ## 5 4 4 3 4 8 9 5 6 6 5 6 6 6 9 -- 6

[H45]46 : ## ## ## ## ## ## ## ## ## ## ## ## ## ## ## ## ## ## ## 5 4 6 4 ## 6 4 ## 5 ## 3 4 4 3 2 6 7 3 4 4 3 4 4 4 7 6 --

COMPUTED FROM THE NETWORK DISTANCE MATRIX

pos: 1 2 3 4 5 6 7 8 9 10 11 12 13 14 15 16 17 18 19 20 21 22 23 24 25 26 27 28 29 30 31 32 33 34 35 36 37 38 39 40 41 42 43 44 45 46

[H1]1 : -- 3 2 3 4 2 3 1 3 2 2 3 2 2 ##

[H2]2 : 3 -- 1 2 3 3 4 2 2 3 3 4 3 3 ##

[H3]3 : 2 1 -- 3 4 2 3 1 1 2 2 3 2 2 ##

[H5]4 : 3 2 3 -- 1 3 4 2 4 3 3 4 3 3 ##

[H4]5 : 4 3 4 1 -- 4 5 3 5 4 4 5 4 4 ##

[H7]6 : 2 3 2 3 4 -- 1 1 3 2 2 3 2 2 ##

[H8]7 : 3 4 3 4 5 1 -- 2 4 3 3 4 3 1 ##

[H6]8 : 1 2 1 2 3 1 2 -- 2 1 1 2 1 1 ##

[H10]9 : 3 2 1 4 5 3 4 2 -- 3 3 4 3 3 ##

[H9]10 : 2 3 2 3 4 2 3 1 3 -- 2 3 2 2 ##

[H11]11 : 2 3 2 3 4 2 3 1 3 2 -- 3 2 2 ##

[H12]12 : 3 4 3 4 5 3 4 2 4 3 3 -- 3 3 ##

[H13]13 : 2 3 2 3 4 2 3 1 3 2 2 3 -- 2 ##

[H14]14 : 2 3 2 3 4 2 1 1 3 2 2 3 2 -- ##

[H16]15 : ## ## ## ## ## ## ## ## ## ## ## ## ## ## -- 1 2 4 2 ## ## ## ## 3 ## ## 8 ## 5 ## ## ## ## ## ## ## ## ## ## ## ## ## ## ## ## ##

[H15]16 : ## ## ## ## ## ## ## ## ## ## ## ## ## ## 1 -- 1 3 1 ## ## ## ## 2 ## ## 7 ## 4 ## ## ## ## ## ## ## ## ## ## ## ## ## ## ## ## ##

[H17]17 : ## ## ## ## ## ## ## ## ## ## ## ## ## ## 2 1 -- 4 2 ## ## ## ## 3 ## ## 8 ## 5 ## ## ## ## ## ## ## ## ## ## ## ## ## ## ## ## ##

[H19]18 : ## ## ## ## ## ## ## ## ## ## ## ## ## ## 4 3 4 -- 4 ## ## ## ## 1 ## ## 4 ## 3 ## ## ## ## ## ## ## ## ## ## ## ## ## ## ## ## ##

[H18]19 : ## ## ## ## ## ## ## ## ## ## ## ## ## ## 2 1 2 4 -- ## ## ## ## 3 ## ## 8 ## 5 ## ## ## ## ## ## ## ## ## ## ## ## ## ## ## ## ##

[H23]20 : ## ## ## ## ## ## ## ## ## ## ## ## ## ## ## ## ## ## ## -- 5 3 5 ## 3 1 ## 6 ## 4 5 5 4 3 5 6 2 5 3 4 3 3 3 6 7 5

[H24]21 : ## ## ## ## ## ## ## ## ## ## ## ## ## ## ## ## ## ## ## 5 -- 6 2 ## 6 4 ## 1 ## 3 4 4 3 2 6 7 3 4 4 3 4 4 4 7 6 4

[H25]22 : ## ## ## ## ## ## ## ## ## ## ## ## ## ## ## ## ## ## ## 3 6 -- 6 ## 4 2 ## 7 ## 5 6 6 5 4 6 7 3 6 4 5 4 4 4 7 8 6

[H26]23 : ## ## ## ## ## ## ## ## ## ## ## ## ## ## ## ## ## ## ## 5 2 6 -- ## 6 4 ## 3 ## 3 4 4 3 2 6 7 3 4 4 3 4 4 4 7 6 4

[H20]24 : ## ## ## ## ## ## ## ## ## ## ## ## ## ## 3 2 3 1 3 ## ## ## ## -- ## ## 5 ## 2 ## ## ## ## ## ## ## ## ## ## ## ## ## ## ## ## ##

[H28]25 : ## ## ## ## ## ## ## ## ## ## ## ## ## ## ## ## ## ## ## 3 6 4 6 ## -- 2 ## 7 ## 5 6 6 5 4 6 7 3 6 4 5 4 4 4 7 8 6

[H27]26 : ## ## ## ## ## ## ## ## ## ## ## ## ## ## ## ## ## ## ## 1 4 2 4 ## 2 -- ## 5 ## 3 4 4 3 2 4 5 1 4 2 3 2 2 2 5 6 4

[H21]27 : ## ## ## ## ## ## ## ## ## ## ## ## ## ## 8 7 8 4 8 ## ## ## ## 5 ## ## -- ## 7 ## ## ## ## ## ## ## ## ## ## ## ## ## ## ## ## ##

[H29]28 : ## ## ## ## ## ## ## ## ## ## ## ## ## ## ## ## ## ## ## 6 1 7 3 ## 7 5 ## -- ## 4 5 5 4 3 7 8 4 5 5 4 5 5 5 8 7 5

[H22]29 : ## ## ## ## ## ## ## ## ## ## ## ## ## ## 5 4 5 3 5 ## ## ## ## 2 ## ## 7 ## -- ## ## ## ## ## ## ## ## ## ## ## ## ## ## ## ## ##

[H30]30 : ## ## ## ## ## ## ## ## ## ## ## ## ## ## ## ## ## ## ## 4 3 5 3 ## 5 3 ## 4 ## -- 3 3 2 1 5 6 2 3 3 2 3 3 3 6 5 3

[H31]31 : ## ## ## ## ## ## ## ## ## ## ## ## ## ## ## ## ## ## ## 5 4 6 4 ## 6 4 ## 5 ## 3 -- 4 3 2 6 7 3 4 4 3 4 4 4 7 6 4

[H33]32 : ## ## ## ## ## ## ## ## ## ## ## ## ## ## ## ## ## ## ## 5 4 6 4 ## 6 4 ## 5 ## 3 4 -- 1 2 6 7 3 4 4 3 4 4 4 7 4 4

[H32]33 : ## ## ## ## ## ## ## ## ## ## ## ## ## ## ## ## ## ## ## 4 3 5 3 ## 5 3 ## 4 ## 2 3 1 -- 1 5 6 2 3 3 2 3 3 3 6 3 3

[H34]34 : ## ## ## ## ## ## ## ## ## ## ## ## ## ## ## ## ## ## ## 3 2 4 2 ## 4 2 ## 3 ## 1 2 2 1 -- 4 5 1 2 2 1 2 2 2 5 4 2

[H35]35 : ## ## ## ## ## ## ## ## ## ## ## ## ## ## ## ## ## ## ## 5 6 6 6 ## 6 4 ## 7 ## 5 6 6 5 4 -- 1 3 4 4 5 4 2 4 1 8 6

[H36]36 : ## ## ## ## ## ## ## ## ## ## ## ## ## ## ## ## ## ## ## 6 7 7 7 ## 7 5 ## 8 ## 6 7 7 6 5 1 -- 4 5 5 6 5 3 5 2 9 7

[H37]37 : ## ## ## ## ## ## ## ## ## ## ## ## ## ## ## ## ## ## ## 2 3 3 3 ## 3 1 ## 4 ## 2 3 3 2 1 3 4 -- 3 1 2 1 1 1 4 5 3

[H38]38 : ## ## ## ## ## ## ## ## ## ## ## ## ## ## ## ## ## ## ## 5 4 6 4 ## 6 4 ## 5 ## 3 4 4 3 2 4 5 3 -- 4 3 4 2 4 5 6 4

[H39]39 : ## ## ## ## ## ## ## ## ## ## ## ## ## ## ## ## ## ## ## 3 4 4 4 ## 4 2 ## 5 ## 3 4 4 3 2 4 5 1 4 -- 3 2 2 2 5 6 4

[H40]40 : ## ## ## ## ## ## ## ## ## ## ## ## ## ## ## ## ## ## ## 4 3 5 3 ## 5 3 ## 4 ## 2 3 3 2 1 5 6 2 3 3 -- 3 3 3 6 5 3

[H43]41 : ## ## ## ## ## ## ## ## ## ## ## ## ## ## ## ## ## ## ## 3 4 4 4 ## 4 2 ## 5 ## 3 4 4 3 2 4 5 1 4 2 3 -- 2 2 5 6 4

[H42]42 : ## ## ## ## ## ## ## ## ## ## ## ## ## ## ## ## ## ## ## 3 4 4 4 ## 4 2 ## 5 ## 3 4 4 3 2 2 3 1 2 2 3 2 -- 2 3 6 4

[H41]43 : ## ## ## ## ## ## ## ## ## ## ## ## ## ## ## ## ## ## ## 3 4 4 4 ## 4 2 ## 5 ## 3 4 4 3 2 4 5 1 4 2 3 2 2 -- 5 6 4

[H44]44 : ## ## ## ## ## ## ## ## ## ## ## ## ## ## ## ## ## ## ## 6 7 7 7 ## 7 5 ## 8 ## 6 7 7 6 5 1 2 4 5 5 6 5 3 5 -- 9 7

[H46]45 : ## ## ## ## ## ## ## ## ## ## ## ## ## ## ## ## ## ## ## 7 6 8 6 ## 8 6 ## 7 ## 5 6 4 3 4 8 9 5 6 6 5 6 6 6 9 -- 6

[H45]46 : ## ## ## ## ## ## ## ## ## ## ## ## ## ## ## ## ## ## ## 5 4 6 4 ## 6 4 ## 5 ## 3 4 4 3 2 6 7 3 4 4 3 4 4 4 7 6 --

DIFFERENCE MATRIX

pos: 1 2 3 4 5 6 7 8 9 10 11 12 13 14 15 16 17 18 19 20 21 22 23 24 25 26 27 28 29 30 31 32 33 34 35 36 37 38 39 40 41 42 43 44 45 46

[H1]1 : -- 0 0 0 0 0 0 0 0 0 0 0 0 0 ##

[H2]2 : 0 -- 0 0 0 0 0 0 0 0 0 0 0 0 ##

[H3]3 : 0 0 -- 0 0 0 0 0 0 0 0 0 0 0 ##

[H5]4 : 0 0 0 -- 0 0 0 0 0 0 0 0 0 0 ##

[H4]5 : 0 0 0 0 -- 1 1 0 0 0 0 0 0 0 ##

[H7]6 : 0 0 0 0 1 -- 0 0 0 0 0 0 0 0 ##

[H8]7 : 0 0 0 0 1 0 -- 0 0 0 0 0 0 0 ##

[H6]8 : 0 0 0 0 0 0 0 -- 0 0 0 0 0 0 ## ## ## ## ## ## ## ## ## ## ## ## ## ##
##

[H10]9 : 0 0 0 0 0 0 0 0 -- 0 0 0 0 0 ## ## ## ## ## ## ## ## ## ## ## ## ## ##
##

[H9]10 : 0 0 0 0 0 0 0 0 0 -- 0 0 0 0 ## ## ## ## ## ## ## ## ## ## ## ## ## ##
##

[H11]11 : 0 0 0 0 0 0 0 0 0 0 -- 0 0 0 ## ## ## ## ## ## ## ## ## ## ## ## ## ##
##

[H12]12 : 0 0 0 0 0 0 0 0 0 0 0 -- 0 0 ## ## ## ## ## ## ## ## ## ## ## ## ## ##
##

[H13]13 : 0 0 0 0 0 0 0 0 0 0 0 0 -- 0 ## ## ## ## ## ## ## ## ## ## ## ## ## ##
##

[H14]14 : 0 0 0 0 0 0 0 0 0 0 0 0 0 -- ## ## ## ## ## ## ## ## ## ## ## ## ## ##
##

[H16]15 : ## ## ## ## ## ## ## ## ## ## ## ## ## ## -- 0 0 0 0 ## ## ## ## 0 ##
0 ## 0 ## ## ## ## ## ## ## ## ## ## ## ## ## ## ## ##

[H15]16 : ## ## ## ## ## ## ## ## ## ## ## ## ## ## 0 -- 0 0 0 ## ## ## ## 0 ##
0 ## 0 ## ## ## ## ## ## ## ## ## ## ## ## ## ## ## ##

[H17]17 : ## ## ## ## ## ## ## ## ## ## ## ## ## ## 0 0 -- 0 0 ## ## ## ## 0 ##
0 ## 0 ## ## ## ## ## ## ## ## ## ## ## ## ## ## ## ##

[H19]18 : ## ## ## ## ## ## ## ## ## ## ## ## ## ## 0 0 0 -- 0 ## ## ## ## 0 ##
0 ## 0 ## ## ## ## ## ## ## ## ## ## ## ## ## ## ## ##

[H18]19 : ## ## ## ## ## ## ## ## ## ## ## ## ## ## 0 0 0 0 -- ## ## ## ## 0 ##
0 ## 0 ## ## ## ## ## ## ## ## ## ## ## ## ## ## ## ##

[H23]20 : ## ## ## ## ## ## ## ## ## ## ## ## ## ## ## ## ## ## ## -- 2 0 2 ## 0
0 ## 2 ## 0 0 0 0 0 0 0 0 0 0 0 0 0 0 0 0 0

[H24]21 : ## ## ## ## ## ## ## ## ## ## ## ## ## ## ## ## ## ## ## 2 -- 4 0 ## 2
2 ## 0 ## 0 0 0 0 0 0 0 0 0 0 0 0 0 0 0 0 0

[H25]22 : ## ## ## ## ## ## ## ## ## ## ## ## ## ## ## ## ## ## ## 0 4 -- 2 ## 0
0 ## 4 ## 0 0 0 0 0 0 0 0 0 0 0 0 0 0 0 0 0

[H26]23 : ## ## ## ## ## ## ## ## ## ## ## ## ## ## ## ## ## ## ## 2 0 2 -- ## 2
2 ## 0 ## 0 0 0 0 0 0 0 0 0 0 0 0 0 0 0 0 0

[H20]24 : ## ## ## ## ## ## ## ## ## ## ## ## ## ## 0 0 0 0 0 ## ## ## ## -- ##
0 ## 0 ## ## ## ## ## ## ## ## ## ## ## ## ## ## ## ##

[H28]25 : ## ## ## ## ## ## ## ## ## ## ## ## ## ## ## ## ## ## ## 0 2 0 2 ## --
0 ## 2 ## 0 0 0 0 0 0 0 0 0 0 0 0 0 0 0 0 0

[H27]26 : ## ## ## ## ## ## ## ## ## ## ## ## ## ## ## ## ## ## ## 0 2 0 2 ## 0
-- ## 2 ## 0 0 0 0 0 0 0 0 0 0 0 0 0 0 0 0 0

[H21]27 : ## ## ## ## ## ## ## ## ## ## ## ## ## ## 0 0 0 0 0 ## ## ## ## 0 ## ## -- ## 0 ## ## ## ## ## ## ## ## ## ## ## ## ## ## ## ## ##

[H29]28 : ## ## ## ## ## ## ## ## ## ## ## ## ## ## ## ## ## ## ## 2 0 4 0 ## 2 2 ## -- ## 0 0 0 0 0 0 0 0 0 0 0 0 0 0 0 0 0 0

[H22]29 : ## ## ## ## ## ## ## ## ## ## ## ## ## ## 0 0 0 0 0 ## ## ## ## 0 ## ## 0 ## -- ## ## ## ## ## ## ## ## ## ## ## ## ## ## ## ## ##

[H30]30 : ## ## ## ## ## ## ## ## ## ## ## ## ## ## ## ## ## ## ## 0 0 0 0 ## 0 0 ## 0 ## -- 0 0 0 0 0 0 0 0 0 0 0 0 0 0 0 0

[H31]31 : ## ## ## ## ## ## ## ## ## ## ## ## ## ## ## ## ## ## ## 0 0 0 0 ## 0 0 ## 0 ## 0 -- 0 0 0 0 0 0 0 0 0 0 0 0 0 2 0

[H33]32 : ## ## ## ## ## ## ## ## ## ## ## ## ## ## ## ## ## ## ## 0 0 0 0 ## 0 0 ## 0 ## 0 0 -- 0 0 0 0 0 0 0 0 0 0 0 0 0 0

[H32]33 : ## ## ## ## ## ## ## ## ## ## ## ## ## ## ## ## ## ## ## 0 0 0 0 ## 0 0 ## 0 ## 0 0 0 -- 0 0 0 0 0 0 0 0 0 0 0 0 0

[H34]34 : ## ## ## ## ## ## ## ## ## ## ## ## ## ## ## ## ## ## ## 0 0 0 0 ## 0 0 ## 0 ## 0 0 0 0 -- 0 0 0 0 0 0 0 0 0 0 0 0

[H35]35 : ## ## ## ## ## ## ## ## ## ## ## ## ## ## ## ## ## ## ## 0 0 0 0 ## 0 0 ## 0 ## 0 0 0 0 0 -- 0 0 0 0 0 0 0 0 0 0 0

[H36]36 : ## ## ## ## ## ## ## ## ## ## ## ## ## ## ## ## ## ## ## 0 0 0 0 ## 0 0 ## 0 ## 0 0 0 0 0 0 -- 0 0 0 0 0 0 1 0 0 0

[H37]37 : ## ## ## ## ## ## ## ## ## ## ## ## ## ## ## ## ## ## ## 0 0 0 0 ## 0 0 ## 0 ## 0 0 0 0 0 0 0 -- 0 0 0 0 0 0 0 0 0

[H38]38 : ## ## ## ## ## ## ## ## ## ## ## ## ## ## ## ## ## ## ## 0 0 0 0 ## 0 0 ## 0 ## 0 0 0 0 0 0 0 0 -- 0 0 0 0 0 0 0 0

[H39]39 : ## ## ## ## ## ## ## ## ## ## ## ## ## ## ## ## ## ## ## 0 0 0 0 ## 0 0 ## 0 ## 0 0 0 0 0 0 0 0 0 -- 0 0 0 0 0 0 0

[H40]40 : ## ## ## ## ## ## ## ## ## ## ## ## ## ## ## ## ## ## ## 0 0 0 0 ## 0 0 ## 0 ## 0 0 0 0 0 0 0 0 0 0 -- 0 0 0 0 0 0

[H43]41 : ## ## ## ## ## ## ## ## ## ## ## ## ## ## ## ## ## ## ## 0 0 0 0 ## 0 0 ## 0 ## 0 0 0 0 0 0 0 0 0 0 0 -- 0 0 0 0 0

[H42]42 : ## ## ## ## ## ## ## ## ## ## ## ## ## ## ## ## ## ## ## 0 0 0 0 ## 0 0 ## 0 ## 0 0 0 0 0 0 0 0 0 0 0 0 -- 0 0 0 0

[H41]43 : ## ## ## ## ## ## ## ## ## ## ## ## ## ## ## ## ## ## ## 0 0 0 0 ## 0 0 ## 0 ## 0 0 0 0 0 0 1 0 0 0 0 0 0 -- 0 0 0

[H44]44 : ## ## ## ## ## ## ## ## ## ## ## ## ## ## ## ## ## ## ## 0 0 0 0 ## 0 0 ## 0 ## 0 0 0 0 0 0 0 0 0 0 0 0 0 0 -- 0 0

[H46]45 : ## ## ## ## ## ## ## ## ## ## ## ## ## ## ## ## ## ## ## 0 0 0 0 ## 0 0 ## 0 ## 0 2 0 0 0 0 0 0 0 0 0 0 0 0 0 -- 0

[H45]46 : ## ## ## ## ## ## ## ## ## ## ## ## ## ## ## ## ## ## ## 0 0 0 0 ## 0 0 ## 0 ## 0 0 0 0 0 0 0 0 0 0 0 0 0 0 0 0 --

The total positive difference matrix is 33.0

The total negative difference matrix is 0.0

Calculations are finished.

____
